# Higher fiber higher carbohydrate diets better than lower carbohydrate lower fiber diets for diabetes management: Rapid review with meta‐analyses

**DOI:** 10.1111/obr.13837

**Published:** 2024-09-19

**Authors:** Andrew N. Reynolds, Jessica Lang, Amanda Brand, Jim Mann

**Affiliations:** ^1^ Edgar Diabetes and Obesity Research Centre (EDOR) University of Otago Dunedin New Zealand; ^2^ Department of Medicine University of Otago Dunedin New Zealand; ^3^ Centre for Evidence‐based Health Care, Division of Epidemiology and Biostatistics, Department of Global Health Stellenbosch University Stellenbosch South Africa

**Keywords:** carbohydrate metabolism, dietary guidelines, meta‐analysis, public health, type 2 diabetes

## Abstract

**Background:**

Some dietary recommendations continue to recommend carbohydrate restriction as a cornerstone of dietary advice for people with diabetes.

**Purpose:**

We compared the cardiometabolic effects of diets higher in both fiber and carbohydrate with lower carbohydrate lower fiber diets in type 1 or type 2 diabetes.

**Data sources:**

MEDLINE, Embase, and the Cochrane Database of Systematic Reviews up to June 24, 2024, with additional hand searching.

**Study selection:**

Randomized controlled trials in which both dietary fiber and carbohydrate amount had been modified were identified from source evidence syntheses on carbohydrate amount in people with diabetes.

**Data extraction:**

Two reviewers independently.

**Data synthesis:**

Ten eligible trials including 499 participants with diabetes (98% with T2) were identified from the potentially eligible 828 trials included in existing evidence syntheses. Pooled findings indicate that higher fiber higher carbohydrate diets reduced HbA1c (mean difference [MD] −0.50% [95% confidence interval −0.99 to −0.02]), fasting insulin (MD −0.99 μIU/mL [−1.83 to −0.15]), total cholesterol (MD −0.16 mmol/L [−0.27 to −0.05]) and low‐density lipoprotein cholesterol (MD −0.16 mmol/L (−0.31 to −0.01) when compared with lower carbohydrate lower fiber diets. Trials with larger differences in fiber and carbohydrate intakes between interventions reported greater reductions. Certainty of evidence for these outcomes was moderate or high, with most outcomes downgraded due to heterogeneity unexplained by any single variable.

**Limitations:**

Our predefined scope excluded trials with co‐interventions such as energy restriction, which may have provided addition information.

**Conclusions:**

Findings indicate the greater importance of promoting dietary fiber intakes, and the relative unimportance of carbohydrate amount in recommendations for people with diabetes.

## 1 INTRODUCTION

Carbohydrates provide around half of global dietary energy intake.^1^ International and national nutrition guidelines indicate that a wide range of carbohydrate intakes are acceptable^2^, ^3^; however, the increasing evidence of the health benefits of dietary fiber^4^ has led to more clearly defined recommendations relating to this subgroup of carbohydrate. In 2023, the World Health Organization (WHO) released perhaps the most decisive of its global dietary guidelines to date, promoting foods rich in dietary fiber to achieve an intake of at least 25 g dietary fiber per day.^3^ While this guideline is consistent with current regional and national recommendations for dietary fiber intake,^5^, ^6^, ^7^ it represents a substantial increase from current global estimated intakes of around 20 g per day.^6^

There is a considerable body of separate evidence regarding the benefits of dietary fiber for people with type 1 and type 2 diabetes, with high‐fiber diets improving glycemic control and a range of cardiovascular risk factors, as well as reducing premature mortality when compared with lower‐fiber diets, in people with diabetes.^8^ Unsurprisingly, dietary guidelines for those with diabetes include recommendations encouraging consumption of fiber‐rich foods and at least 35 g dietary fiber per day.^2^ However, there has been far less consistency regarding advice relating to the amount of total carbohydrate intake in diabetes management. The discovery of insulin in the 1920s enabled those with type 1 diabetes to metabolize carbohydrate. Before that time starvation diets or diets with little or no carbohydrate were seen as the only management tool available. Despite the absence of evidence of long‐term benefit of carbohydrate restriction^9^, ^10^, ^11^, ^12^ advice promoting low‐carbohydrate diets has remained a fairly consistent feature of dietary guidelines for diabetes albeit with caveats to their use,^13^, ^14^ despite a lack of consensus on what a low‐carbohydrate diet is.^15^ Current contraindications to low‐carbohydrate diets include being pregnant, lactating, childhood, renal disease, risk of disordered eating, and sodium‐glucose cotransporter inhibitor (SGLT2) use.^13^

To date, there has been no published synthesis regarding the extent to which increasing dietary fiber and carbohydrate intakes together may influence cardiometabolic risk factors for people with diabetes. We have done these analyses to clarify dietary advice relating to both fiber and total carbohydrate intakes as it currently stands, and move towards consistent evidence‐based dietary advice from authoritative bodies in diabetes management.

## 2 RESEARCH DESIGN AND METHODS

We have conducted a rapid review to identify the existing evidence syntheses (such as systematic reviews) of carbohydrate intake in diabetes management, to be used as source documents for eligible trials. We did this rather than search for trials directly given the number of systematic reviews already available on carbohydrate intake. We then went through each evidence synthesis to identify eligible randomized controlled trials of dietary interventions where prescribed intakes of both carbohydrate and fiber differed by a priori‐determined minimum amounts in one intervention compared with another. We then performed meta‐analyses with the relevant trials. Our methods were informed by Cochrane methodology for conducting reviews and meta‐analyses.^16^ We used the PRISMA reporting standards for systematic reviews and meta‐analyses^17^ to guide our reporting. The protocol for this review was prospectively registered on PROSPERO (CRD42023473322).

### 2.1 Literature search

The online search strategy for the rapid review included a term for study design (i.e. “systematic review”), combined with a term for exposure (i.e. “carbohydrate”) and a term for population group (i.e. “diabetes”). Full search terms used, and terms considered in sensitivity testing, alongside the population, intervention, comparator, and outcome (PICO) format inclusion criteria, are shown in the Supporting Information. Ovid MEDLINE, Embase, and the Cochrane Database of Systematic Reviews were searched up to June 24, 2024. The online search was augmented by hand searching reference lists and bibliographies of identified evidence syntheses and included trials to identify other potentially eligible publications. No date or language restrictions were applied to the searches. Commercially available software was used to remove duplicates and aid screening (Covidence, Veritas Health Innovation). Two reviewers screened all titles, abstracts, and full texts independently and in duplicate to identify eligible publications. Disagreements in screening were discussed until consensus was reached.

### 2.2 Randomized controlled trial eligibility

To identify evidence relevant to our question, we extracted data from trials included in eligible source syntheses to assess whether these trials were eligible for new meta‐analyses. We considered controlled trials reporting on participants with type 1 or type 2 diabetes receiving interventions of interest for at least 6 weeks to be eligible. The diet in the intervention arms of trials needed to differ from the diet in the control arm by a minimum 5% in total energy (TE) intake derived from carbohydrates, and had to have a concomitant difference in dietary fiber intake of at least 3.5 g per day per 5%TE carbohydrate difference. Three and a half grams of dietary fiber per 5% change in TE from carbohydrates was chosen as the minimum threshold based on recent quantitative recommendations^2^ for fiber intake in diabetes management (at least 35 g per day), and a global average of around 50% dietary energy provided from carbohydrates.^6^ As this value is not based on an expected physiological threshold of effect, we undertook several methods of testing the fiber increase relative to the carbohydrate increase. How the carbohydrate and fiber differences between intervention arms were generated did not influence trial eligibility (e.g., by supplementation, macronutrient intake advice, or broader dietary pattern advice). Eligible trials included those in which participants were provided with foods or were given dietary advice. Both parallel and crossover trials, with or without a washout period, were eligible. Trials that did not achieve the prespecified difference in carbohydrate intakes between intervention arms, or where the differences could not be calculated, were excluded. We included only dietary composition interventions as eligible, excluding those trials with additional components such as advice to reduce energy intake or increase physical activity. Prespecified outcomes of interest were those used in clinical management of diabetes and related to glycemic control (e.g., primary outcome glycated hemoglobin [HbA1C], fasting plasma or serum glucose, and fasting insulin), anthropometry (body weight and body mass index [BMI]), blood lipids (total cholesterol, low‐density and high‐density lipoprotein [LDL and HDL] cholesterol, cholesterol ratio, and triglycerides), and blood pressure (systolic and diastolic).

### 2.3 Data extraction and risk of bias assessment

Data from source evidence syntheses and eligible trials were extracted by one reviewer into an Excel spreadsheet template used in a previous review,^18^ with a second reviewer checking every value extracted. For source evidence syntheses, descriptive data were extracted; this included a summary of their pooled findings and the Altmetric score of each review. For eligible trials, both descriptive data and those required for quantitative synthesis in our new meta‐analyses were extracted. For our meta‐analyses, we prioritized the extraction of (1) pre‐ and post‐intervention measures when stated, (2) difference per intervention when stated, (3) post‐intervention only values when stated, or (4) values obtained from a Webplot digitizer when results were only displayed in graphical format. We standardized outcomes reported in different units of measurement with widely accepted formulas (e.g., mg/dL to mmol/L for lipids). We converted confidence intervals to standard deviations, and standard deviations to standard errors when necessary.^16^ Authors of eligible trials were contacted to provide additional details when necessary.

Source evidence syntheses were not critically appraised for risk of bias as they did not need to be for the purposes of this rapid review. Risk of bias of individual trials eligible to for the meta‐analyses were extracted from source evidence syntheses when they stated that this assessment was undertaken by at least two reviewers independent of each other (Cochrane risk of bias tool preferentially extracted).

### 2.4 Statistical analyses

For our updated meta‐analyses of controlled trials, we analyzed the mean difference (MD) between intervention arms with generic inverse variance models and random effects, as we anticipated the presence of heterogeneity between studies, to quantify the effect of higher fiber higher carbohydrate diets in diabetes management. All prespecified outcomes of interest were continuous variables. For trials with more than one eligible comparison, we have avoided a unit of analysis error by splitting the participant number in the common comparator arm.^16^ Correlation coefficients were obtained from publications when reported, or taken from a previous review with a larger pool of trials on diabetes management.^8^ The proportion of heterogeneity of pooled results that is due to true variance in effects was assessed with the *I* ^2^ statistic.^19^ Sensitivity analyses were conducted on all pooled analyses. First, the effect of each individual study on the pooled result was considered with an influence analysis. This involved the removal of intervention data from the pooled estimate one at a time. Small study effects, as might be seen with publication bias, were assessed with Egger's test^20^ and the trim‐and‐fill method.^21^ Potential mediators of the differences in outcomes between trials, and hence drivers of heterogeneity, were extensively considered with meta‐regression analyses. Prespecified meta‐regression variables related to study design characteristic (i.e., parallel or crossover), participant characteristics (i.e., type 1 or type 2 diabetes and medication use), trial characteristics (i.e., intervention duration and risk of bias), and comparison characteristics (i.e., size of the carbohydrate or fiber difference between interventions and the type of diet prescribed to generate carbohydrate differences). The “Results” section presents the overall pooled results, and every subgrouping of relevant trials when (and only when) meta‐regression analyses indicated that variable influenced the pooled result. We used the ICEMAN criteria to comment on the credibility of effect modification from these analyses.^22^ All forest plots and meta‐regression testing are shown in the Supporting Information. Analyses are undertaken in Stata (Version 17) with the *metan*, *metaninf*, *metabias*, *meta*, and *metareg* commands.

The evidence generated was considered with Grading of Recommendations Assessment, Development and Evaluation (GRADE) protocols to comment on the certainty of evidence for higher fiber higher carbohydrate diets in diabetes management. The GRADEPro (GRADEpro GDT, McMaster University and Evidence Prime) software was used to help assess evidence certainty.

## 3 RESULTS

### 3.1 Evidence syntheses

**FIGURE 1:**
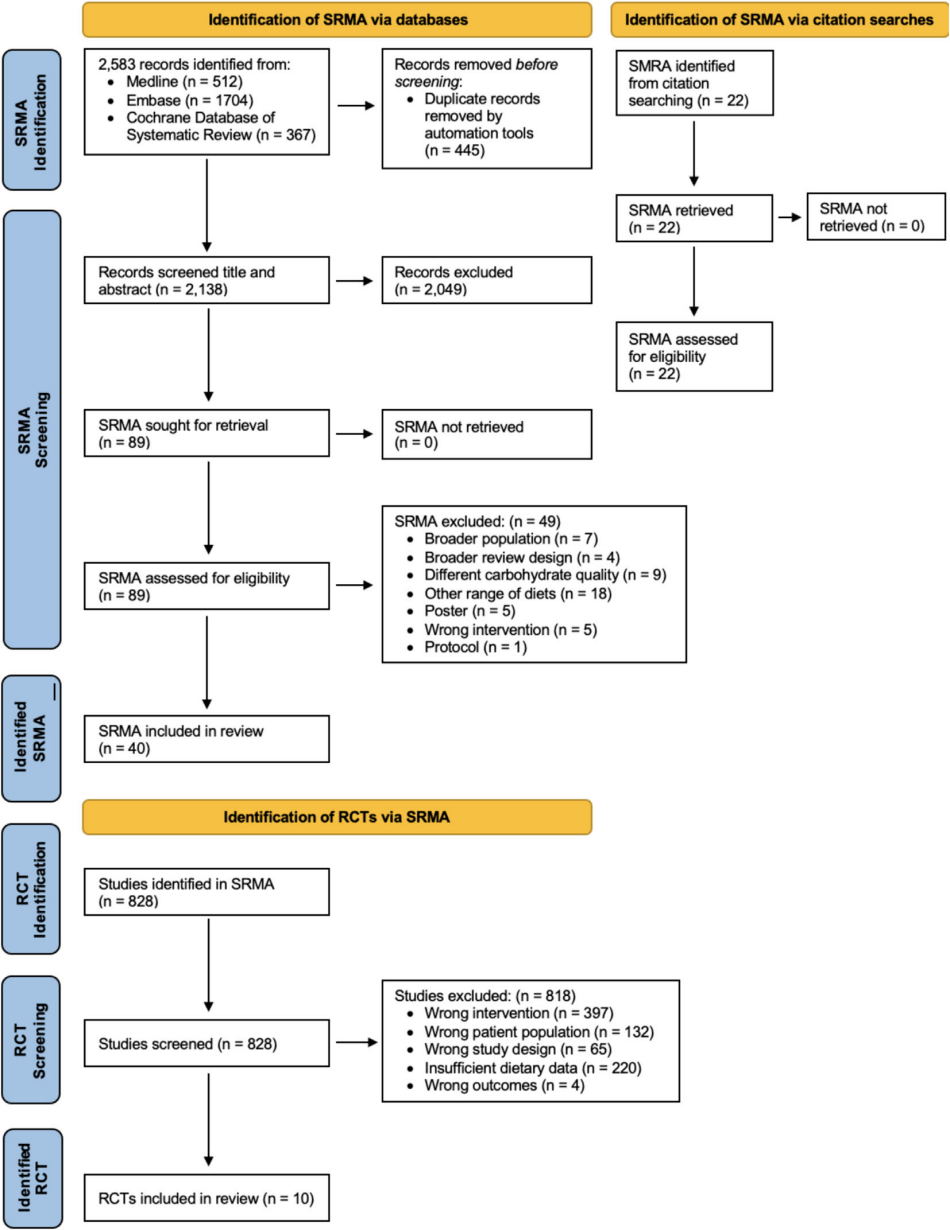
Flowchart illustrating the identification of eligible evidence syntheses and trials.

The PRISMA flowchart outlining the identification of eligible evidence syntheses and then randomized controlled trials is shown in Figure 1. We identified 40 evidence syntheses that specifically reported on trials moderating carbohydrate amount in diabetes management, and a further 49 evidence syntheses of broader study designs (i.e., umbrella reviews), participant populations (i.e., including those without diabetes), and interventions (i.e., different carbohydrate parameters or multiple dietary interventions) relevant to our research question. Details of each identified review are shown in Tables S1 and S2.

### 3.2 Trials

**FIGURE 2:**
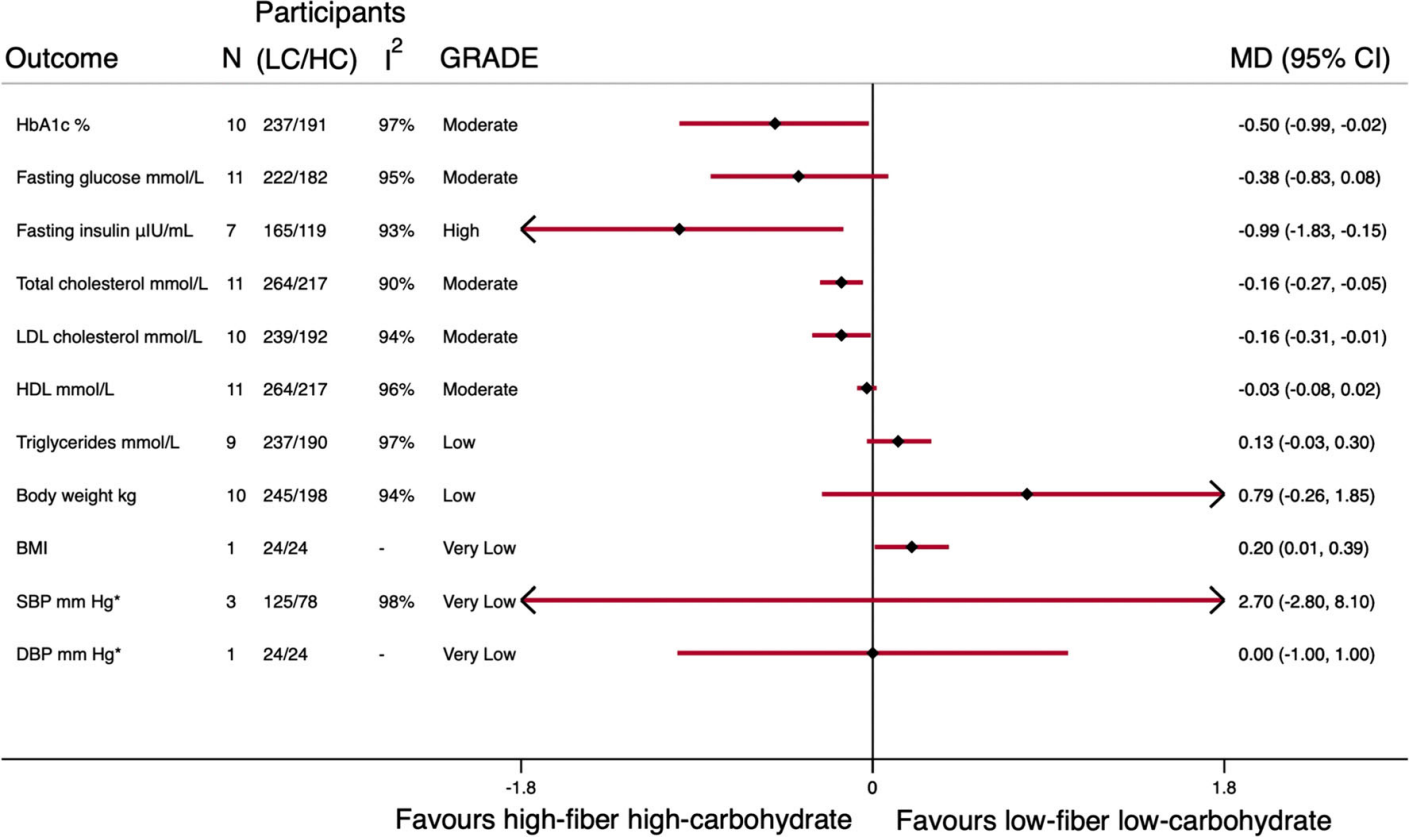
Higher‐carbohydrate higher‐fiber diets compared with lower‐carbohydrate lower‐fiber diets in diabetes management. *One trial that provided different instruction on sodium intake between intervention arms was removed before analysis of these outcomes.

From the 89 evidence syntheses, we identified 828 unique studies, 10 of which met our eligibility criteria. One trial had three relevant intervention arms enabling two comparisons. Descriptive details of eligible trials are shown in Table 1. Trials included 499 participants with diabetes, and were conducted in the UK,^24^, ^25^, ^26^, ^27^, ^28^, ^31^ Europe,^23^ North America,^29^, ^32^ and Australia.^30^ Only nine participants had type 1 diabetes, the remaining 490 had type 2 diabetes. The difference in carbohydrate as a percentage of TE between interventions of eligible trials ranged from 5.4% to 28%, and the difference in fiber consumed between interventions ranged from 6.4 to 85 g per day. The lower carbohydrate arm ranged from 34–47%TE (fiber 12.7–35.5 g/day), and the higher carbohydrate arm ranged from 49–65%TE (fiber 21–100 g/day). Higher fiber higher carbohydrate diets were achieved by the promotion of: foods high in fiber; foods high in fiber and unsaturated fats; or low glycemic index (GI) diets. Conversely, lower carbohydrate lower fiber diets focused on unsaturated fat intakes, lower carbohydrate intakes, or lower fiber intakes. Only three trials of 186 participants used a supplement to increase fiber intakes; the remaining seven trials did so through food provision or advice. The pooled effects of higher fiber higher carbohydrate diets are shown in Figure 2. Subgroups discussed in text are every instance where a dichotomous meta‐regression indicated that a difference between trials may be influencing the pooled result. All forest plots, meta‐regression results, and GRADE tables are shown in Tables S3–S5.

**TABLE 1:** Description of the 10 identified trials included in meta‐analyses.

Publication	N trial participants (*n* female)	Intervention duration	Lower CHO arm(s) (CHO%TE and fiber g/day)	Higher CHO arm (CHO%TE and fiber g/day)	Outcomes measured
Bozzetto (2012), Italy^23^	45^8^	8 weeks	Advice to consume a high monounsaturated fat diet (40% and 18.4 g/day)	Advice to consume a high carbohydrate, high fiber, low GI diet (53% and 48.7 g/day). Included fiber supplement use	HbA1c Body weight Fasting plasma glucose Fasting plasma insulin Total cholesterol HDL cholesterol LDL cholesterol Triglycerides Fasting plasma insulin
Dodson (1984), England^24^	50^25^	12 weeks	Advice to consume a modern Western diet (47% and 20 g/day)	Advice to consume high unrefined carbohydrate, high fiber diet (53% and 40–45 g/day). Included fiber supplement use	HbA1c Body weight Total cholesterol HDL cholesterol Triglycerides Systolic blood pressure Diastolic blood pressure
Frost (1994), England^26^	60^15^	12 weeks	Standard advice was based on the 1982 British Diabetic Association dietary recommendations (44% and 14 g/day)	Advice to consume a low GI diet (49% and 21 g/day)	Body weight Fasting plasma glucose Total cholesterol HDL cholesterol LDL cholesterol Triglycerides
Lousley (1983), England^25^	15 (0)	6 weeks each diet (crossover)	Advice to consume a low carbohydrate diet (37% and 12.7 g/day)	Advice to consume a high carbohydrate high fiber diet (65% and 67.7 g/day)	HbA1c Body weight Fasting plasma glucose Fasting plasma insulin Total cholesterol HDL cholesterol LDL cholesterol Triglycerides
Simpson (1979), England^27^	18^3^	6 weeks each diet (crossover)	Advice to consume a low carbohydrate diet (34% and 35.5 g/day)	Advice to consume a high carbohydrate‐modified fat diet (61% and 78 g/day)	HbA1c Fasting plasma glucose Total cholesterol HDL cholesterol LDL cholesterol
Simpson (1981), England^28^	18 NIDDM^8^ 9 IDDM^5^	6 weeks each diet (crossover)	Advice to consume a low carbohydrate diet (40% and 17.6 g/day)	Advice to consume a high carbohydrate diet containing leguminous and fiber (61% and 96.6 g/day)	HbA1c Fasting plasma glucose Fasting plasma insulin Total cholesterol HDL cholesterol LDL cholesterol Triglycerides
Tsihlias (2000), Canada^29^	91 (43)	24 weeks	Advice to consume a high monounsaturated fat diet (43.2% and 23.5 g/day)	Advice to consume a low GI diet (50.1% and 50.3 g/day). Included fiber supplement use	HbA1c Body weight Fasting plasma glucose Total cholesterol HDL cholesterol LDL cholesterol Triglycerides Cholesterol ratio
Walker (1995), Australia^30^	24^15^	12 weeks each diet (crossover)	Advice to consume a modified fat diet (40% and 25 g/day)	Advice to consume a high carbohydrate low fat diet (50% and 34 g/day)	HbA1c Body weight BMI Fasting plasma glucose Fasting plasma insulin Total cholesterol HDL cholesterol LDL cholesterol Triglycerides Systolic blood pressure Diastolic blood pressure
Ward (1982), England^31^	7 (0)	6 weeks each diet (crossover)	Advice to consume a low carbohydrate diet (40% and 15 g/day)	Advice to consume a high carbohydrate high fiber diet (60% and 100 g/day)	Body weight Fasting plasma glucose Fasting plasma insulin
Wolever (2008, Canada^32^	162 (162)	52 weeks	Advice to consume a low carbohydrate diet (39.3% and 23 g/day) Or (two comparisons available) Advice to consume a high GI diet (46.5% and 21 g/day)	Advice to consume a low GI diet (51.9% and 36.3 g/day)	HbA1c Body weight Fasting plasma glucose Fasting plasma insulin Total cholesterol HDL cholesterol LDL cholesterol Triglycerides Systolic blood pressure

Abbreviations: BMI, body mass index; CHO, carbohydrate; g/day, grams per day; GI, glycaemic index; Hba1c, glycated haemoglobin; HDL, high‐density lipoprotein; IDDM, insulin dependent diabetes melitus; LDL, low‐density lipoprotein; N, number; NIDDM, non‐insulin dependent diabetes melitus; TE, total energy.

### 3.3 Glycemic control and insulin levels

**FIGURE 3:**
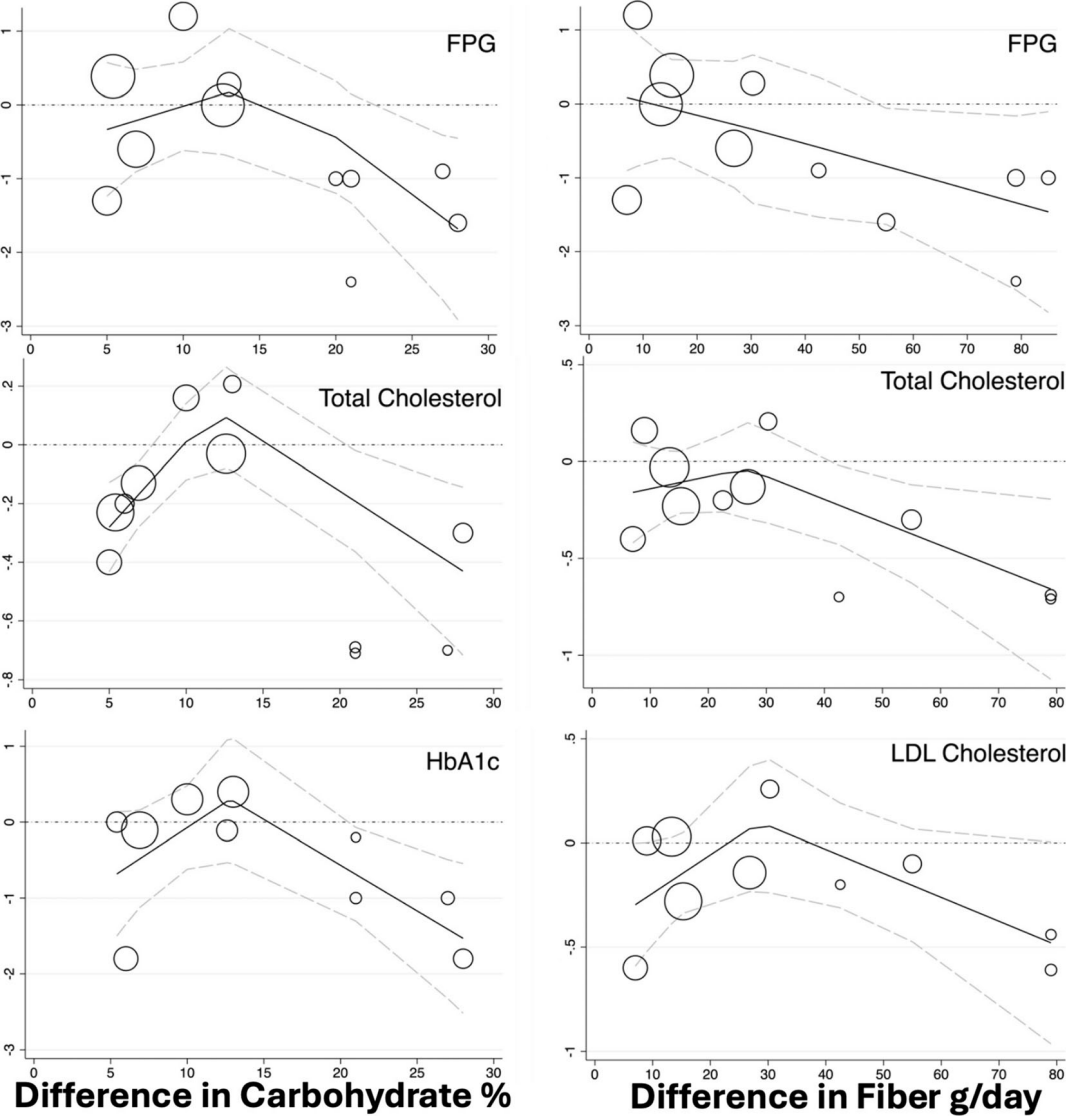
Dose response testing for carbohydrate and fiber difference between intervention arms.

For the primary outcome, higher fiber higher carbohydrate diets reduced HbA1c by −0.50% (95% CI −0.01 to −0.99%) on average, equivalent to −5.5 mmol/mol (95% CI −0 to −11 mmol/mol). This pooled estimate was robust, with continuous meta‐regression of %TE from carbohydrates indicating the greater the difference in carbohydrate amount between intervention arms, the greater the improvement in HbA1c. This is confirmed by dichotomous meta‐regression that identified the MD of four trials where the control arm promoted low carbohydrate intakes (MD −1.07% 95% CI −1.86 to −0.28) was greater than the six trials where the control arm did not actively promote lower carbohydrate intakes (MD 0.16% [95% CI −0.12 to 0.44]). The pooled findings for fasting glucose trended towards improvement but were not significant; however, continuous meta‐regressions identified that the higher the carbohydrate and the higher the fiber different between intervention arms, the larger the improvement in fasting glucose (Figure 3). The certainty of evidence for both HbA1c and fasting glucose was downgraded once to moderate certainty as no single meta‐regression accounted for the high initial heterogeneity of the pooled evidence. The pooled estimate for fasting insulin was robust (Figure 2). Although the initial heterogeneity was high at *I* ^2^ = 93%, a meta‐regression on the fiber increase relative to the carbohydrate increase identified a difference between the pooled trials. The three trials with the lowest fiber to carbohydrate increase had a pooled MD 0.02 μIU/mL (95% CI −0.65 to 0.69) with an *I* ^2^ of 18%. The four trials with a higher fiber to carbohydrate increase had a pooled MD −1.40 μIU/mL (95% CI −1.62 to −1.18) and *I* ^2^ of 0%. Because of the broad nature of the interventions in the trials identified, and the identification of an important variable and the resulting low heterogeneity of the subgroup analyses, this outcome was not downgraded for inconsistency resulting in high‐certainty evidence.

### 3.4 Blood lipids

Pooled effects for both total cholesterol and LDL cholesterol indicated reductions with higher fiber higher carbohydrate diets. There was evidence from continuous meta‐regressions that reduction in these outcomes was greater in trials generating the largest differences in the carbohydrate (for total cholesterol) and fiber (for total and LDL cholesterol) between intervention arms (Figure 3). For total cholesterol, in four trials where participants in the lower carbohydrate trial arm received advice to consume unsaturated fats, dichotomous meta‐regression of the pooled effect did not show a difference between trial arms (MD 0.00 mmol/L (95% CI −0.20 to 0.20). In comparison, there were appreciable improvements in total cholesterol in the higher fiber higher carbohydrate arm for the seven trials where participants in the lower carbohydrate arm did not receive this advice (MD −0.29 mmol/L (95% CI −0.44 to −0.13). Similarly, there was a difference for LDL cholesterol in the eight trials where the lower carbohydrate arm received mixed interventions (MD −0.04 mmol/L (95% CI −0.14 to 0.07) when compared with the two trials where participants in the lower carbohydrate arm received advice to consume simple or low‐fiber carbohydrates (MD −0.44 mmol/L [95% CI −0.75 to −0.12]). The pooled data for HDL cholesterol and triglycerides were largely robust and did not indicate significant differences when following higher fiber higher carbohydrate diets (Figure 2). The certainty of evidence for all lipid outcomes were downgraded once for inconsistency as no single meta‐regression explained the initial high heterogeneity, with triglycerides being downgraded a further time for imprecision.

### 3.5 Anthropometry and blood pressure

The pooled evidence for body weight indicated both the approach used to increase carbohydrate and fiber content and medication use greatly influenced this pooled result. The trials where the intervention directly targeted high intakes of whole grains and legumes indicated a reduction in body weight (MD −0.85 kg [95% CI −2.65 to 0.95 kg]), when compared with trials that focused on lower GI (MD 1.73 kg [95% CI 0.53 to 2.93]) or high fiber and healthy fat intakes (MD 0.59 kg [95% CI 0.01 to 1.16]), which both indicated increases in body weight. In a separate dichotomous meta‐regression, seven trials where eligible participants could be on anti‐hyperglycemic medication reported a different result (MD −0.25 kg [95% CI −1.49 to 0.99]) from that observed in the three trials in participants not on anti‐hyperglycemic drug therapy (MD 2.61 kg [95% CI 1.83 to 3.38 kg]). It should be noted that these three trial comparisons were the low GI diet trials, potentially confounding this finding. There were insufficient data to provide informed comment on the role of higher fiber higher carbohydrate diets on BMI and blood pressure in diabetes management (very low‐certainty evidence).

## 4 DISCUSSION

Overall, we identified that higher fiber higher carbohydrate intakes are likely beneficial to patients with diabetes when compared with lower carbohydrate lower fiber diets through the reduction of HbA1c, fasting insulin, total cholesterol, and LDL cholesterol. While several different dietary approaches were used to achieve the contrast between diets, meta‐regression analyses identified logical drivers of heterogeneity, indicating a robust data set. Greater improvements in outcome measures were observed with larger differences in carbohydrate and fiber amount. Further analyses taking into account the nature of the dietary interventions used to achieve the high and low carbohydrate intakes revealed differences of potential clinical relevance. While the total pool of evidence indicated improvements in total and LDL cholesterol, when the lower carbohydrate intervention arm focused on increasing unsaturated fat intakes, there were unsurprisingly no differences. Interestingly, the dietary approach used to generate fiber and carbohydrate differences between interventions appeared to moderate the effect on body weight. Diets promoting whole grains and legumes indicated a reduction in body weight (MD −0.85 kg [−2.65 to 0.95]), while diets of high fiber and healthy fat intakes (0.59 kg [0.01 to 1.16]) and lower GI diets (1.73 kg [0.53 to 2.93]) appreciably increased body weight, in line with recent data from prospective observational studies.^33^

Our findings are congruent with previous meta‐analyses and enable clarification of several aspects of dietary advice where uncertainty remains. Previous evidence synthesis has identified that high fiber diets improve cardiometabolic risk factors as well as reduce premature mortality for people with type 1 and type 2 diabetes.^8^ This larger body of trials identified a consistent HbA1c reduction of 2.0 mmol/mol (95% CI 0.7 to 3.3) when fiber intake had been increased,^8^ comparable with the current finding equivalent to 6 mmol/mol (95% CI 0 to 12) HbA1c reduction. Similar findings on the benefits of higher fiber intakes have been reported in those with cardiovascular disease, hypertension, and in the general population.^4^, ^18^ Conversely, several meta‐analyses have shown that any improvement in cardiometabolic risk factors with low‐ or very low‐carbohydrate diets are fleeting and not maintained in the long term.^9^, ^10^, ^11^, ^12^ A comprehensive Cochrane systematic review of trials, published in 2022, established a non‐significant reduction (MD −0.14% [95% CI −0.38 to 0.10]) for HbA1c with low‐carbohydrate weight reducing diets when compared with balanced carbohydrate weight‐reducing diets in participants with type 2 diabetes and overweight or obesity with a weight‐reducing phase lasting 12 months or longer.^10^ In stark contrast to our findings on higher fiber higher carbohydrate diets, that review concluded that there is “probably little to no difference between lower and higher carbohydrate diets for changes in heart disease risks, like diastolic blood pressure, glycosylated and LDL cholesterol up to two years.” Taken together, there remains no clear justification to focus a reduction in total carbohydrate intake for those with diabetes, as some current guidelines do,^13^, ^14^ especially when doing so may result in a concurrent reduction in fiber intakes.

Future research can provide further nuanced information on the role of carbohydrates in blood glucose control. It is notable that the most recent trial in the current analyses was published in 2012, over a decade ago. This topic is worth more attention and research, with newer data able to provide more nuanced findings and better credibility of the effect modification observed with meta‐regression.^22^ Future research could consider high‐fiber low‐carbohydrate diets. Trials of high‐fiber low‐carbohydrate diets could be identified from our rapid review. These diets could be compared with low‐fiber low‐carbohydrate diets, either using within‐trial comparisons or across trial comparisons by meta‐regression. Further, more social science–based research is needed to understand why low‐carbohydrate messages may be so pervasive in current culture and for some health professionals, despite the evidence of their limited efficacy.^9^, ^10^, ^11^, ^12^ Low‐carbohydrate diets may be incorrectly promoted due to their initial use in type 1 diabetes management over 100 years ago, or they may have other traits that make them appealing to promote. Low‐carbohydrate diets may be higher‐meat diets, which may have a social desirability aspect as meat products are generally more expensive than carbohydrate‐based foods, and therefore may be a status symbol for some. Following a low‐carbohydrate diet may also imply self‐restriction or restraint, broadcasting a strong will or determination to others. Further social determinants may also drive people towards accepting low‐carbohydrate diets as beneficial despite the evidence base. Future research towards understanding these aspects and our own human nature may provide pathways to dispel unsupported dietary behaviors.

The methodology employed and novel consideration of trials that have manipulated both carbohydrate amount and fiber content are major strengths of our study. To date, no evidence synthesis has considered the trials where both carbohydrate amount and fiber have been manipulated. We conducted a rapid review to identify the evidence syntheses of all trials involving modification of carbohydrate amount in people with diabetes, and then, using standardized criteria undertook a meta‐analysis of those trials which enabled a comparison between diets which were relatively high in both total carbohydrate and dietary fiber and those with lower intakes. A limitation was that only 10 eligible trials including 499 participants were identified, of which only 2% had T1. However, despite the limited evidence base, we observed consistency in findings and were able to readily explain the nature of the identified variables that influenced pooled results, such as trials of higher fiber differences having greater improvements in cardiometabolic risk factors. The data available were from high‐income countries only, and only three trials used fiber supplements reducing our ability to conduct robust analyses to consider potential differences between intrinsic, extracted, and synthetic fibers in diabetes management. We have considered trials with at least 5%TE difference in carbohydrate intake between intervention arms, rather than discretize this variable into categories of “low” or “very low” where consensus definitions do not exist.^15^ While many of the lower carbohydrate arms identified might not meet the various definitions of “low” or “very low” carbohydrate (range 34–47%TE), there is also no evidence of a threshold effect on cardiometabolic risk factors from trials that would justify use of such categories. The narrow scope of this review, as presented in its prospective registration, considers only trials without co‐interventions may also be considered as a limitation. Trials which included physical activity interventions or energy reduction may have provided addition information, especially when co‐interventions were balanced between arms.^23^, ^34^

These new analyses, alongside the existing evidence and guidelines, indicate that nutrition guidance should not focus on carbohydrate amount, but carbohydrate type and source instead. Dietary fiber intakes for adults with diabetes should be at least 35 g per day, through the intake of minimally processed carbohydrate sources: whole grains, legumes, vegetables, and fruit.^2^ Intakes of added sugars should be below 10% TE,^35^ with sugars intakes derived from minimally processed foods such as fruits rather than added sugars like those found in sugar sweetened beverages, or highly processed foods.

## AUTHOR CONTRIBUTIONS

ANR designed the study, undertook the searches, checked the data extraction, undertook the analyses, and wrote the first draft of the manuscript. JL undertook the searches, extracted the data, and reviewed and edited the manuscript. AB designed the study, informed the analyses and write up, and reviewed and edited the manuscript. JIM designed the study, informed the analyses and write up, and reviewed and edited the manuscript. ANR is the guarantor of this work and, as such, had full access to all the data in the study and takes responsibility for the integrity of the data and the accuracy of the data analysis. All authors approved the final version of the manuscript.

## CONFLICT OF INTEREST STATEMENT

The authors declare no conflicts of interest.

## Supporting information


**Table S1:** Identified systematic reviews and meta analyses reporting on trials moderating carbohydrate amount in diabetes management.
**Table S2:** Identified systematic reviews and meta analyses reporting on trials of broader carbohydrate parameters in diabetes management.
**Table S3:** Forest plots per outcome.
**Table S4:** Meta‐regression analyses per outcome.
**Table S5:** GRADE Tables.

## Data Availability

Sourced from the original publications listed in manuscript and supplemental material, or provided by authors in personal correspondence.
