# Supplementary material for: Higher fiber higher carbohydrate diets better than lower carbohydrate lower fiber diets for diabetes management: Rapid review with meta‐analyses
Source: Obes Rev. 2024 Sep 19;26(1):e13837. doi: 10.1111/obr.13837 (PMC11611436; doi:10.1111/obr.13837)
Supplement: Supplementary file 1 — Table S1: Identified systematic reviews and meta analyses reporting on trials moderating carbohydrate amount in diabetes management. Table S2: Identified systematic reviews and meta analyses reporting on trials of broader carbohydrate parameters in diabetes management. Table S3: Forest plots per outcome. Table S4: Meta‐regression analyses per outcome. Table S5: GRADE Tables. [file OBR-26-e13837-s001.pdf]

**Supplemental Material for:** Higher fibre higher carbohydrate diets better than lower carbohydrate lower fibre diets for diabetes management: rapid review with meta-analyses

Andrew N Reynolds[1,2], Jessica Lang[2], Amanda Brand[3] & Jim Mann[1,2]

1. Edgar Diabetes and Obesity Research Centre (EDOR), University of Otago, New Zealand.
2. Department of Medicine, University of Otago, New Zealand.
3. Centre for Evidence-based Health Care, Division of Epidemiology and Biostatistics, Department of Global Health, Stellenbosch University, South Africa

**Contact information:** andrew.reynolds@otago.ac.nz

**Supplemental Material 1**

***PICO for systematic reviews***
*Participants* with type 1 or type 2 diabetes.
*Interventions* of lower or higher carbohydrate intake (i.e. as a % of total energy).
*Comparisons* between trials included in source evidence synthesis, such as systematic reviews.
*Outcomes* relevant to diabetes management

***PICO for randomised controlled trials***
*Participants* with type 1 or type 2 diabetes.
*Interventions* were of at least six weeks duration and generated a minimum difference in total energy (TE) intake derived from carbohydrates and concomitant difference in dietary fibre intake of 3.5g per day or higher per 5%TE carbohydrate difference between intervention arms. Trials with additional intervention exposures (i.e. hypocaloric) will be excluded. Trials where the difference in carbohydrate and fibre cannot be calculated will also be excluded.
*Comparato*r The effects of higher-fibre higher-carbohydrate diets compared with lower-fibre lower-carbohydrate diets.
*Outcomes* of interest are differences between interventions in glycaemic control (e.g., primary outcome glycated haemoglobin (HbA1C), fasting plasma or serum glucose), fasting insulin, anthropometry (body weight and body mass index (BMI)), blood lipids (total cholesterol, low-density and high-density lipoprotein (LDL and HDL) cholesterol, triglycerides), and blood pressure (systolic and diastolic).

**Online search term development for systematic review.**
**Term for exposure**: Carbohydrate OR fibre OR fiber Or non-starch polysaccharide OR non starch polysaccharide OR dietary fibre

Exposure terms considered and dropped due to non-specificity: NSP, CHO, Dietary fibre

**Term for population group**: diabet* OR prediabet* or T1* OR T2* OR NIDDM OR type 1 diabetes OR type 2 diabetes OR IDDM OR non-insulin dependent OR non insulin dependent OR insulin dependent OR adult onset diabetes OR adult-onset diabetes OR juvenile-onset diabetes OR juvenile onset diabetes

**Term for study design**: systematic review  OR Meta analysis OR meta-analysis OR meta analyses OR meta-analyses

**Supplemental Table 1: Identified systematic reviews and meta analyses reporting on trials moderating carbohydrate amount in diabetes management**

| First author, year of publication (reference) | No. of DBs searched | No. of RCTs included | No. of participants | Conclusion | Funding | Altmetric score at 10.04.2024 |
|---|---|---|---|---|---|---|
| Abbasnezhad, 2020 [1] | 4 DBs: PubMed, the Cochrane Library, Web of Science, and Scopus | 16 | 1610 | Favours high monounsaturated fat diet over high carbohydrate diets | Did not receive any specific grant from funding agencies in the public, commercial, or not-for-profit sectors | 2 |
| Alarim, 2020 [2] | 1 DB: ovid | 4 | 178 | Favours ketogenic diet for glycaemic control and blood lipids | Not reported | 218 |
| Amini, 2024 [3] | 4 DBs: PubMed/Medline, SCOPUS, Cochrane Library, and Google Scholar | 23 | 1164 | Conclusion states no consistent differences in blood pressure with ketogenic diets | Not reported | 27 |
| Anderson, 2004 [4] | Not reported | 12 | 180 | Favours moderate to high carbohydrate (55% of TE) and high fibre diet (25-50 g/day) | Funded by the Heart and Stroke Foundation of Canada, NSERC Canada, Unilever, Loblaw Brands Ltd., National Starch, Protein Technologies, Almond Board of California, International Nut Council, Quaker, Ceapro and Hain-Celestial Group | 31 |
| Apekey, 2022 [5] | 5 DBs: PubMed, MEDLINE, Embase, Cochrane Library, Web of Science, Clinical Trials.gov | 22 | 1391 | Results favours low carbohydrate for reducing HbA1c levels and adiposity parameters at short-to-intermediate terms. Conclusion states low carbohydrate diet equally effective as a low fat diet in obese patients with T2D | Received no external funding | 40 |
| Castaneda-Gonzalez, 2011 [6] | 3 DBs: PubMed, The Cochrane library and EBSCOhost | 8 | 664 | Conclusion states no consistent differences in weight and A1C with different diets | Not reported | 8 |

| | | | | | | |
|---|---|---|---|---|---|---|
| Choi, 2023 [7] | 4 DBs: MEDLINE (via PubMed), EMbase, Cochrane, and KoreaMed | 50 | 6834 | Favours a moderately low carbohydrate diet, very-low carbohydrate not recommended | Funded by the National Academy of Medicine of Korea | 1 |
| Choy, 2023 [8] | 5 DBs: PubMed, MEDLINE, Embase, Cochrane Library and CINAHL | 11 | 541 | Favours ketogenic diet for lipid profiles but no additional benefits for glycaemic control or weight loss compared with control diet | No funding received | 21 |
| Dyson, 2020 [9] | 3 DBs: MEDLINE, EMBASE and the Cochrane Central Register of Controlled Trials | 2 | 109 | A lack of high-quality evidence limits the use of very low carbohydrate ketogenic diets in people with diabetes | Not reported | 1 |
| Fan, 2016 [10] | 4 DBs: PubMed, Medline, Embase, and Cochrane Library | 10 | 1080 | Favours low carbohydrate diet for weight and HbA1c | Not reported | NA |
| Goldenberg, 2021 [11] | 5 DBs: CENTRAL, Medline, Embase, CINAHL, CAB | 23 | 1357 | Favours low carbohydrate diet for remission of diabetes without adverse consequences | Funded in part by Texas A&M University | 1104 |
| Hernandez Alcantara, 2015 [12] | 3 DBs: PubMed, EBSCOhost and Scielo | 4 | 444 | Conclusion states equivalence with other diets | In Spanish | 2 |
| Huntriss, 2018 [13] | 7 DBs: MEDLINE, EMBASE, CINAHL, Cochrane Central Register of Controlled Trials, ISRCTN, ProQuest and opengrey.eu | 18 | 2204 | Reducing dietary carbohydrate may produce clinical improvements in the management of type 2 diabetes | Funded by the National Institutes of Health | 200 |
| Jayedi, 2022 [14] | 3 DBs: PubMed, Scopus, and Web of Science | 50 | 4291 | Favours low carbohydrate diet for cardiometabolic risk factors in diabetes management | No funding received | 546 |
| Jooste, 2023 [15] | 4 DBs: Medline, Embase, CINAHL, and Web of Science | 2 | 125 | Inconclusive | Received no external funding | 3 |

| | | | | | | |
|---|---|---|---|---|---|---|
| Kirk, 2008 [16] | 5 DBs: PubMed, CINAHL, the Combined Health Information Database, the Cochrane Library and Web of Science | 9 | 187 | Short-term restricted-carbohydrate diets may improve glycaemic control and triglyceride levels in patients with type 2 diabetes | Funded by the National Institutes of Health | 10 |
| Kodama, 2009 [17] | 2 DBs: MEDLINE and the Cochrane Central Register of Controlled Trials (CENTRAL) | 19 | 306 | Favours high fat low carbohydrate diet for insulin resistance | Funded by the Japan Society for the promotion of Science and the Japan Cardiovascular Research Foundation | 15 |
| Korsmo-Haugen, 2019 [18] | 6 DBs: MEDLINE, EMBASE, CENTRAL, CINAHL, Food Science Source and SweMed+ | 23 | 2178 | No benefit with low carbohydrate diet in diabetes management | No funding received | 116 |
| Li, 2022 [19] | 4 DBs: PubMed, Cochrane Library, Embase, and Web of Science | 10 | 717 | Favours a very low carbohydrate ketogenic diet for lipid profile | Not reported | 18 |
| Li, 2021 [20] | 5 DBs: PubMed, Ovid, Embase databases, Cochrane Central Register of Controlled Trials (CENTRAL), and ClinicalTrials.gov | 12 | 761 | Favours low carbohydrate diet over low fat diet in diabetes management | Funded by the National Natural Science Foundation of China, Beijing Natural Science Foundation, Beijing Municipal Science and Technology Commission, National Key Research and Development Program of China and Chinese Academy of Medical Sciences Innovation Fund for Medical Sciences | 29 |
| Luo, 2022 [21] | 5 DBs: PubMed, EMBASE, Web of Science, OVID, and Cochrane Library databases | 21 | 980 | Favours low carbohydrate ketogenic diet in diabetes management | Not reported | 93 |
| McArdle, 2019 [22] | 3 DBs: Medline, EMBASE and CINAHL | 25 | 2132 | Limited benefit on Hba1c in trials up to six months with low carbohydrate diet | Funded by the National Institute for Health Research and Health Education England | 63 |

| | | | | | | |
|---|---|---|---|---|---|---|
| Meng, 2017 [23] | 3 DBs: MEDLINE, EMBASE, and Cochrane Library | 9 | 734 | Favours low carbohydrate diet in diabetes management | Funded by the National Natural Science Foundation of China | 249 |
| Nicholas, 2021 [24] | 5 DBs: Medline, EMBASE, CINAHL, Scopus and Cochrane Central Register of Controlled Trials (CENTRAL) | 15 | 1831 | Low carbohydrate unrestricted energy intake show similar benefits to restricted energy diets for diabetes management | Not reported | 42 |
| Parry-Strong, 2022 [25] | 6 DBs: Medline (OVID), Embase (OVID), Scopus, EBM Reviews—Cochrane Central Register of Controlled Trials (Ovid) and Web of Science | 8 | 606 | Inconclusive conclusions could not show advantage of low carbohydrate diets over other strategies | Funded by the University of Otago | 24 |
| Rafiullah, 2022 [26] | 4 DBs: Ovid MEDLINE, Ovid Embase, CENTRAL, and CINAHL | 8 | 648 | Very low carbohydrate diets superior in diabetes management | Received no external funding | 58 |
| Sainsbury, 2018 [27] | 5 DBs: Medline, Embase, Cumulative Index to Nursing and Allied Health (CINAHL), Global Health and Cochrane Central Register of Controlled Trials (CENTRAL) | 25 | 2412 | Favours low carbohydrate diet for hba1c in diabetes management | This research did not receive any specific grant from funding agencies in the public, commercial, or not-for-profit sectors | 75 |
| Silverii, 2020 [28] | 4 DBs: PubMed, Cochrane, Clinical Trials.gov, and Embase | 37 | 3301 | Favours low carbohydrate diets in the short term for diabetes management | This research was performed as a part of the institutional activity of the unit, with no specific funding | 30 |
| Skow, 2023 [29] | 2 DBs: Directory of Open Access Journals and PubMed | 9 | 670 | Favours ketogenic diets in diabetes management | No funding received | 44 |

| | | | | | | |
|---|---|---|---|---|---|---|
| Snorgaard, 2017 [30] | 3 DBs: Cochrane library databases, EMBASE, and MEDLINE | 10 | 1376 | Favours low carbohydrate diets in diabetes management for the first year. | This research received no specific grant. OS received grant from the Danish Health Authority as the primary author of the Danish National Clinical Guideline for lifestyle intervention in type 2 diabetes | 350 |
| Stamati, 2023 [31] | 2 DBs: Pubmed and Cochrane library | 9 | 143 | Favours low carbohydrate diet in the short term for diabetes management | This research did not receive any specific grant from funding agencies in the public, commercial or not-for-profit sectors. | 0 |
| Tinguely, 2021 [32] | 3 DBs: PubMed, Embase, and the Cochrane Central database | 8 | 477 | Ketogenic diet improves hba1c in diabetes management | Funded by the Université de Lausanne | 70 |
| Turton, 2018 [33] | 4 DBs: MEDLINE; CINAHL; Cochrane Library; and EMBASE | 2 | 20 | Does not draw a conclusion for low carbohydrate diets in type 1 management | No funding received | 119 |
| Turton, 2019 [34] | 5 DBs: MEDLINE, Pre-MEDLINE, EMBASE, CINAHL and the Cochrane Library of Controlled Trials | 18 | 494 | Low carbohydrate diets can be safe in type 2 management | Funded by the University of Sydney | 202 |
| Valenzuela Mencia, 2017 [35] | 7 DBs: Medline, Scopus, Cinahl, Lilacs, Dialnet, Scielo and ProQuest | 11 | 1218 | Favours low carbohydrate diets in the short term | In Spanish | 0 |

| | | | | | | |
|---|---|---|---|---|---|---|
| Van Zuuren, 2018 [36] | 12 DBs: Medline, PubMed, Embase, Web of Science, Cochrane Library, Cochrane Central Register of Controlled Trials (CENTRAL), Emcare, Academic Search Premier, ScienceDirect, Latin American and Caribbean Health Science Information database (LILACS), and Índice Bibliográfico Español en Ciencias de Salud (IBECS) | 33 | 2103 | "Currently available data provide low- to moderate certainty evidence that dietary carbohydrate restriction to a maximum of 40% yields slightly better metabolic control of uncertain clinical importance than reduction in fat to a maximum of 30% in people with T2D" | Funded by the Dutch Diabetes Foundation and Sanofi. | 251 |
| Yuan, 2020 [37] | 2 DBs: PubMed and MEDLINE | 13 | 567 | Favours ketogenic diet in diabetes management | This study was supported by the Science Technology Department of Jilin Province, the Interdisciplinary Project of First Hospital of Jilin University, the Transformation Project of First Hospital of Jilin University and the Education Department of Jilin Province | 497 |
| Zaki, 2022 [38] | 6 DBs: ScienceDirect, Google Scholar, PubMed, Scopus, Embase, and Web of Science | 10 | 747 | Favours ketogenic diet in diabetes management | No funding received | 4 |
| Zaki, 2022 [39] | 5 DBs: PubMed, ScienceDirect, Embase, Web of Science, and Google Scholar | 14 | 1257 | Favours ketogenic diet as more effective than low carbohydrate diet | No funding received | 7 |

| | | | | | | |
|---|---|---|---|---|---|---|
| Zhou, 2022 [40] | 4 DBs: PubMed, Embase, Web of Science, and Cochrane | 8 | 538 | Favours ketogenic diet in diabetes management | Funded by the National Natural Science Foundation of China and the Fundamental Research Funds for the Central Universities as well as the Chutian Scholar Program and Innovative Start-Up Foundation from Wuhan Sports University to N.C | 35 |

**Supplemental Table 2: Identified systematic reviews and meta analyses reporting on trials of broader carbohydrate parameters in diabetes management**

| First author, year of publication (reference) | N databases searched | N included RCTs | N included participants | Reason for exclusion | Altmetric score at 10.04.2024 |
|---|---|---|---|---|---|
| Abboud, 2021 [41] | 7 BDs: PubMed, APA PsycInfo, EMBASE, the Cumulative Index to Nursing and Allied Health Literature (CINAHL), the Cochrane Library, and Clinicaltrials.gov | 9 | 566 | Broader population (adults with chronic disease) | 30 |
| Abbasnezhad, 2020 [42] | 4 DBs: Web of Science, PubMed, Scopus and Cochrane library | 24 | 1130 | Multiple range of dietary patterns | 2 |
| Aghnezhad 2024 [43] | 2 DBs: Pubmed and Medline | Not reported | Not reported | Poster | NA |
| Ajala, 2013 [44] | 3 DBs: PubMed, Embase, and Google Scholar | 20 | 3073 | Multiple range of dietary patterns | 423 |
| Bajorek, 2010 [45] | 4 DBs: PubMed, Cochrane Library, The Natural Standard, and The Natural Medicines | 7 | 386 | Different carbohydrate quality (dietary fibre and low GI diets) | 22 |
| Bierbaum, 2015 [46] | 2 DBs: PubMed and ScienceDirect | 10 | 1845 | Multiple range of dietary patterns | NA |
| Bonekamp, 2023 [47] | 5 DBs: PubMed, Embase, the Cochrane library, SCOPUS and Web of Science | 73 | 5753 | Multiple range of dietary patterns | 37 |
| Buccino, 2004 [48] | Not reported | 31 | Not reported | Multiple range of dietary patterns | NA |
| Castaneda-Gonzalez, 2011 [49] | 3 DBs: PubMed, The Cochrane library and EBSCOhost | 8 | 664 | Poster | NA |
| Choi, 2020 [50] | 3 DBs: Embase, PubMed, and Cochrane Library | 14 | 734 | Broader population (adults with or without diabetes) | 392 |

| | | | | | |
|---|---|---|---|---|---|
| Churuangsuk, 2022 [51] | 4 DBs: MEDLINE (Ovid), PubMed, Web of Science and Cochrane Database of Systematic Reviews | 217 | 18543 | Broader review design (Umbrella review) and multiple dietary patterns | 505 |
| Dening, 2020 [52] | 4 DBs: Medline, Embase, The Cochrane Library, and CINAHL | 5 | 1056 | Wrong intervention (web based interventions for dietary behaviour) | 20 |
| Emadian, 2015 [53] | 4 DBs: Medline, Embase, Cinahl and Web of Science | 11 | 1266 | Multiple range of dietary patterns | 17 |
| Franz, 2010 [54] | 3 DBs: PubMed MEDLINE, the Database of Abstracts of Reviews of Effects, and the Agency for Healthcare Research and Quality | 30 | 3166 | Wrong intervention (medical nutrition therapy) | 35 |
| Harrington, 2011 [55] | Not reported | 10 | 340 | Poster and other range of diets | NA |
| Jing, 2023 [56] | 4 DBs: PubMed, Embase, Web of Knowledge, Cochrane Central Register of Controlled Trials (CENTRAL) | 42 | 4809 | Multiple range of dietary patterns | 45 |
| Jirapinyo, 2018 [57] | 3 DBs: MEDLINE, EMBASE and Web of Science | 16 | 1595 | Poster and other range of diets | NA |
| Johnson, 2010 [58] | 7 DBs: EMBASE, CINAHL®, Cochrane Library, Medline®, PASCAL, PsycINFO®, and Sociological Abstracts | 0 | N/A | Multiple range of dietary patterns | 1 |
| Lu, 2023 [59] | 4 DBs: PubMed, Embase, Web of Science, and Cochrane library | 17 | 642 | Different carbohydrate quality (soluble dietary fibre) | 1 |
| Maula, 2020 [60] | 5 DBs: CINAHL, MEDLINE, Embase, Scopus and the Cochrane Central Register of Controlled Trials (CENTRAL) | 49 | 12461 | Wrong intervention (educational weight loss interventions) | 22 |
| Mcardle, 2017 [61] | Not reported | 24 | 2445 | Poster | NA |

| | | | | | |
|---|---|---|---|---|---|
| Naude, 2014 [62] | 3 DBs: Medline, EMBASE and CENTRAL | 19 | 3209 | Broader population (adults with chronic disease) | 418 |
| Naude, 2022 [63] | 6 DBs: MEDLINE (PubMed), Embase (Ovid), the Cochrane Central Register of Controlled Trials (CENTRAL), Web of Science Core Collection (Clarivate Analytics), ClinicalTrials.gov and WHO International Clinical Trials Registry Platform (ICTRP) | 61 | 6925 | Broader population (adults with chronic disease) | 624 |
| Neuenschwander, 2019 [64] | 2 DBs: Pubmed and the Cochrane Central Register of Controlled Trials (CENTRAL) | 52 | 5360 | Multiple range of dietary patterns | 17 |
| Nield, 2007 [65] | 5 DBs: The Cochrane Library, MEDLINE, EMBASE, CINAHL, AMED | 18 | 1467 | Multiple range of dietary patterns | 16 |
| Nitzke, 2024 [66] | 3 DBs: Scopus, PubMed, and Web of Science | 26 | 2975 | Different carbohydrate quality (high-fibre diets) | NA |
| Ojo, 2018 [67] | 7 DBs: EBSCoHost research, Academic Search Premier, Medline, the Psychology and Behavioural Sciences Collection, PSYCInfo, Cumulative Index to Nursing and Allied Health Literature (CINAHL) and pubmed | 9 | 705 | Different carbohydrate quality (low GI) | 72 |
| Pan, 2019 [68] | 3 DBs: PubMed, EMBASE, and the Cochrane Central Register of Controlled Trials (CENTRAL) | 10 | 921 | Multiple range of dietary patterns | 49 |
| Papamichou, 2019 [69] | 3 DBs: Cochrane CENTRAL, PubMed and Scopus databases | 20 | 2657 | Multiple range of dietary patterns | 66 |
| Pavlidou, 2023 [70] | 3 DBs: PubMed, Cochrane, and Embase | 7 | 1394 | Broader population (adults with or without diabetes) | 21 |

| | | | | | |
|---|---|---|---|---|---|
| Reynolds, 2019 [71] | 4 DBs: PubMed, Ovid MEDLINE, Embase, and the Cochrane Central Register of Controlled Trials | 58 | 4635 | Broader population (normoglycemic only) | 4168 |
| Reynolds, 2020 [72] | 4 DBs: OVID Medline, Embase, PubMed, and the Cochrane Central Register of Controlled Trials | 42 | 1789 | Different carbohydrate quality (dietary fibre and wholegrains) | 605 |
| Ross, 2021 [73] | 5 DBs: Ovid Medline, The Cochrane Library, CINAHL and Embase | 8 | 1217 | Broader population (adults with chronic disease) | 4 |
| Schwingshackl, 2017 [74] | 3 DBs: CENTRAL, PubMed, Google Scholar | N/A | N/A | Protocol | 2 |
| Schwingshackl, 2018 [75] | 3 DBs: PubMed, Cochrane CENTRAL, and Google Scholar | 56 | 4937 | Multiple range of dietary patterns | 146 |
| Szczerba, 2023 [76] | 4 DBs: PubMed, Embase, Cochrane and Epistemonikos | Not reported | Not reported | Broader review design (umbrella review) | 45 |
| Snelson, 2019 [77] | 5 DBs: Ovid MEDLINE, Scopus, CINAHL, Embase and Web of Science | 22 | 670 | Broader population ( adults with and without chronic disease) and different carbohydrate quality (Resistant Starch Type 2) | 17 |
| Swedish Council on Health Technology, 2010 [78] | 3DBs: PubMed, EMBASE and Cochrane Library | 23 | 7001 | Multiple range of dietary patterns and broader population (includes prediabetics) | 9 |
| Swedish Council on Health Technology, 2022 [79] | 4 DBs: MEDLINE (Ovid), PubMed, Web of Science and Cochrane Database of Systematic Reviews | 57 | 39,303 | Multiple range of dietary patterns | 0 |
| Wheeler, 2012 [80] | 1 DB: pubmed | 39 | 1941 | Multiple range of dietary patterns | 64 |
| Whiteley, 2023 [81] | 4 DBs: MEDLINE (Ovid), PubMed, Web of Science and Cochrane Database of Systematic Reviews | 212 | 29590 | Broader review design (Umbrella review) and multiple dietary patterns | 9 |

| | | | | | |
|---|---|---|---|---|---|
| Wolfram, 2011 [82] | 3 DBs: PubMed, Medline, and Google Scholar | 14 | 756 | Different carbohydrate quality (high-fibre diets) | 71 |
| Xie, 2021 [83] | 4 DBs: MEDLINE, Embase, Web of Science, ClinicalTrials.gov, and Cochrane databases | 29 | 1517 | Different carbohydrate quality (soluble fibre supplementation) | 3 |
| Xu, 2021 [84] | 3 DBs: MEDLINE, EMBASE, and CINAHL | 45 | 1995 | Broader review design (umbrella review) | 25 |
| Xu, 2021 [85] | 4 DBs: Web of Science, Pubmed, Scopus and Cochrane library | 16 | 1068 | Different carbohydrate quality (whole grains) | 22 |
| Xu, 2023 (3) | 4 DBs: PubMed, EMBASE, Web of Science, and ScienceDirect | 12 | 676 | Wrong intervention (improving gut microbiota) | 2 |
| Yamada, 2018 [86] | 3 DBs: MEDLINE, EMBASE, and Japan Medical Abstracts Society | 3 | 105 | Multiple range of dietary patterns | 41 |
| Zakarneh, 2023 [87] | 3 DBs: MEDLINE PubMed, ScienceDirect, and Embase | Not reported | Not reported | Multiple range of dietary patterns | 0 |
| Zeng, 2023 [88] | 12 DBs: PubMed, Web of Science, Embase, CINAHL and Open Dissertation, ProQuest, Scopus, Global Index Medicus, Cochrane Central Register of Controlled Trials, Clinicaltrials.gov, SinoMed, WanFang Med, and CNKI | 107 | 8909 | Multiple range of dietary patterns and broader population (includes prediabetics) | 2 |
| Zhang, 2023 [89] | 5 DBs: PubMed, CINAHL, Embase, Web of Science Core Collection, and Cochrane Library | 7 | 3785 | Wrong intervention (effectiveness of lifestyle interventions for diabetes remission) | 8 |

**Supplemental Table 3: Forest plots per outcome**

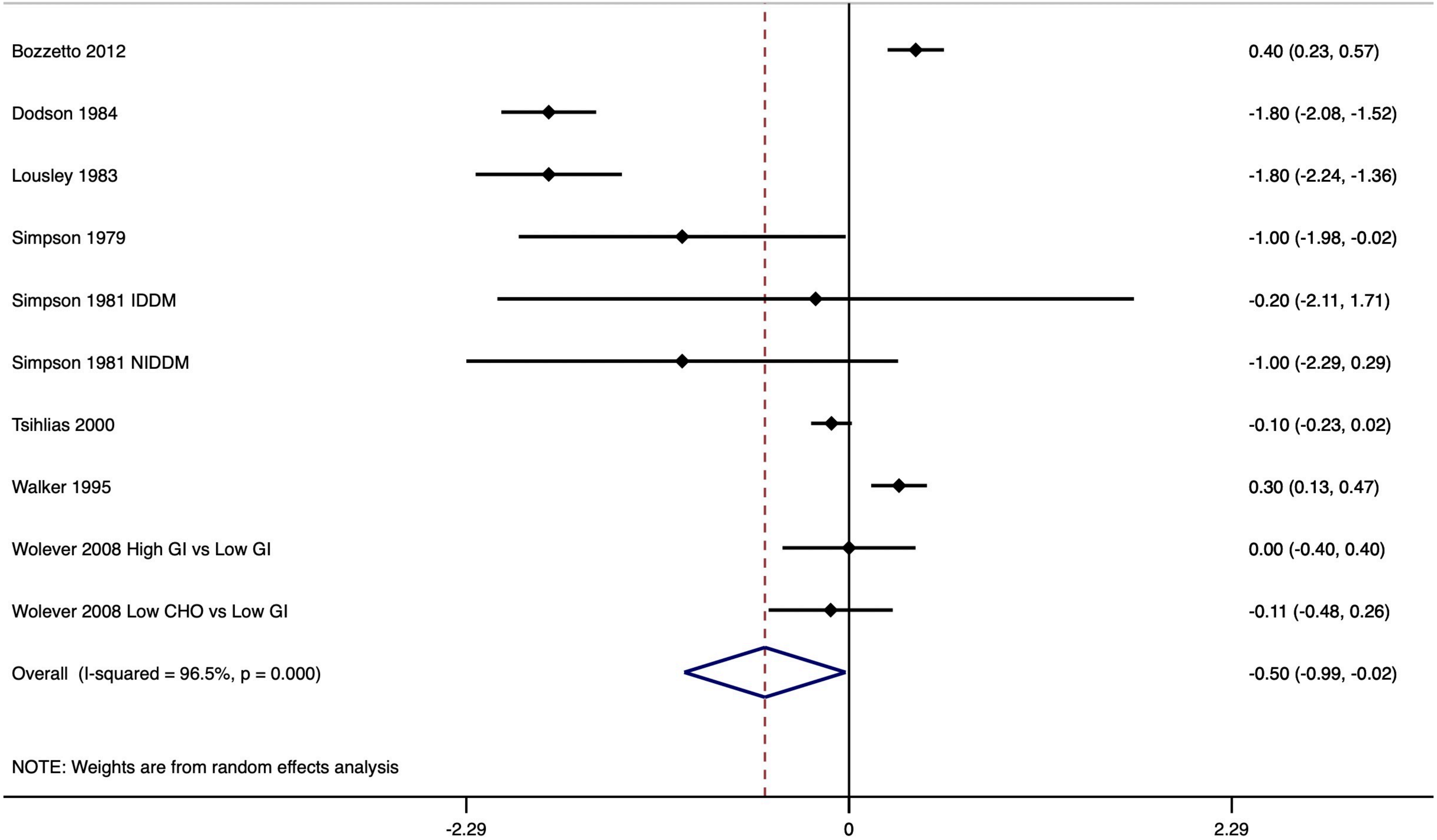


**3.1 HbA1c (%)**

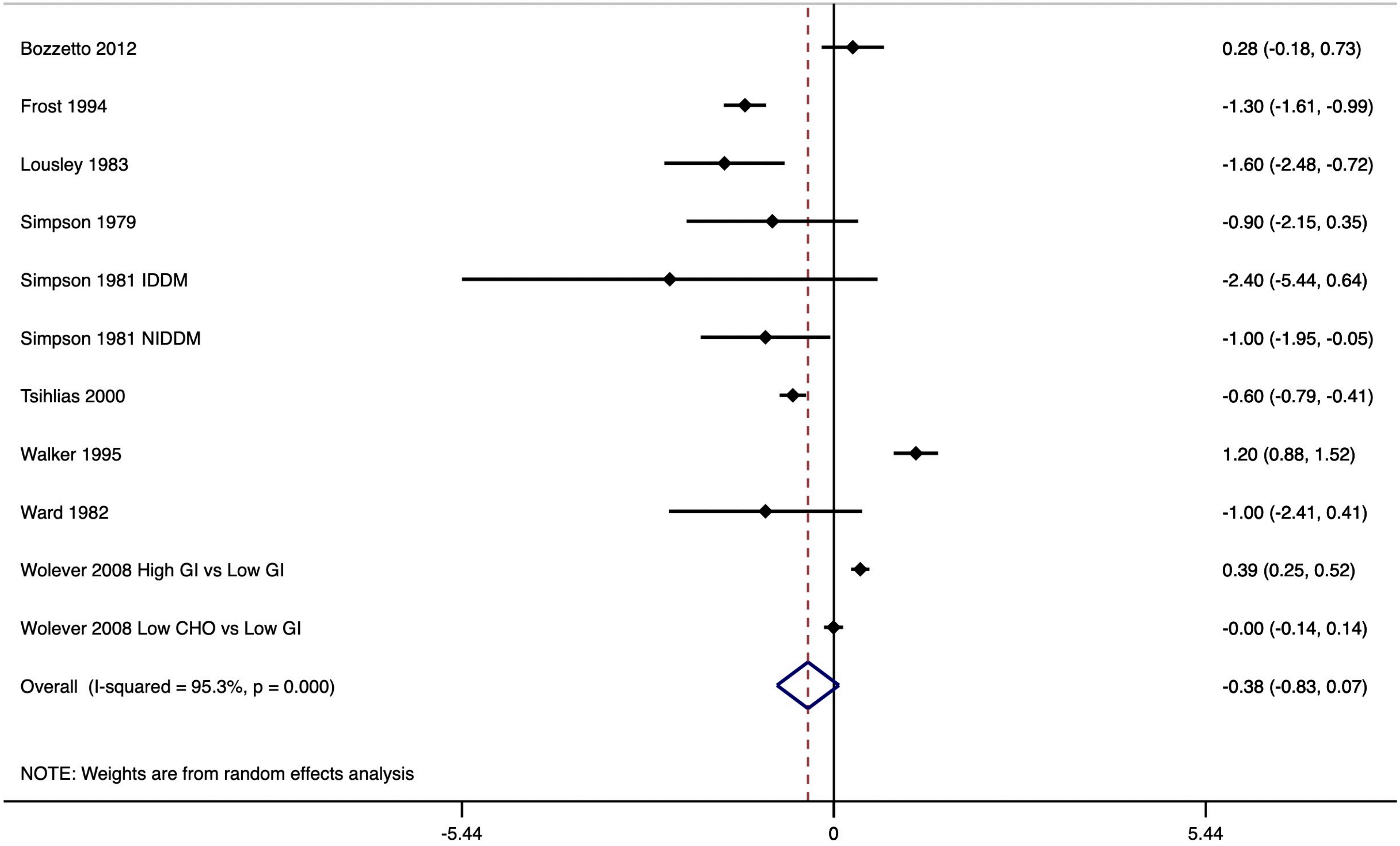


**3.2 Fasting glucose (mmol/L)**

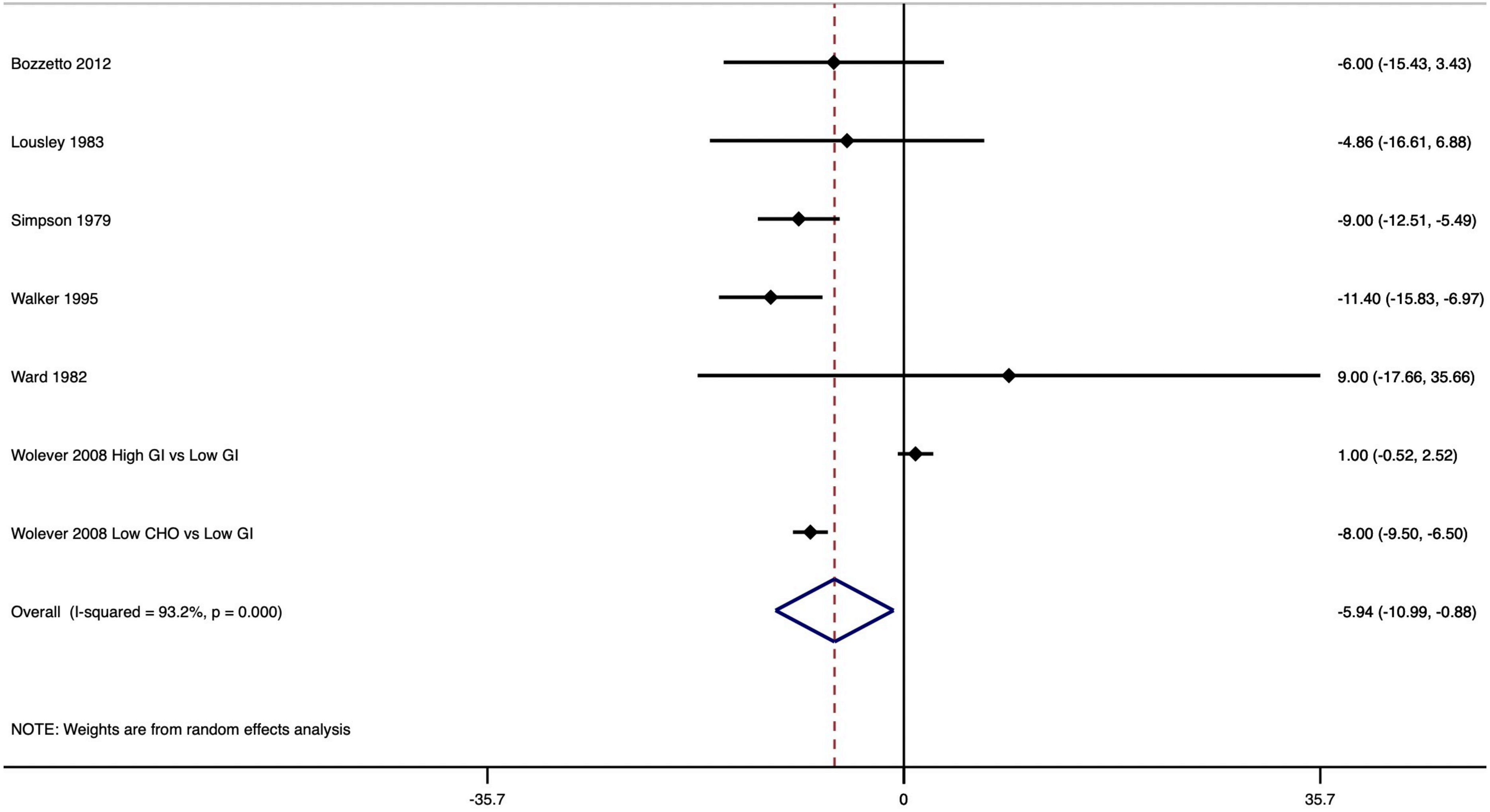


**3.3 Fasting insulin pmol/L**

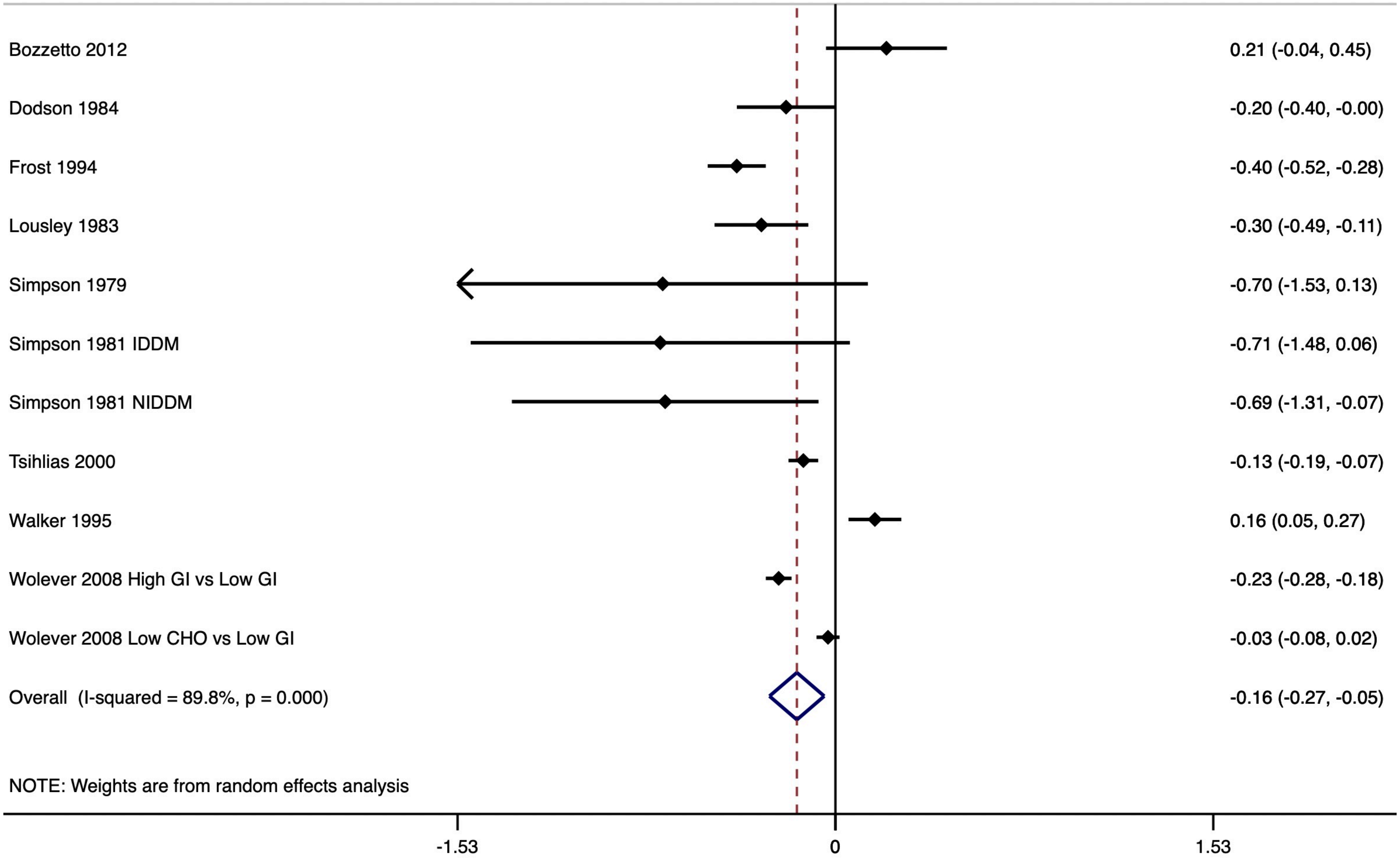


**3.4 Total cholesterol mmol/L**

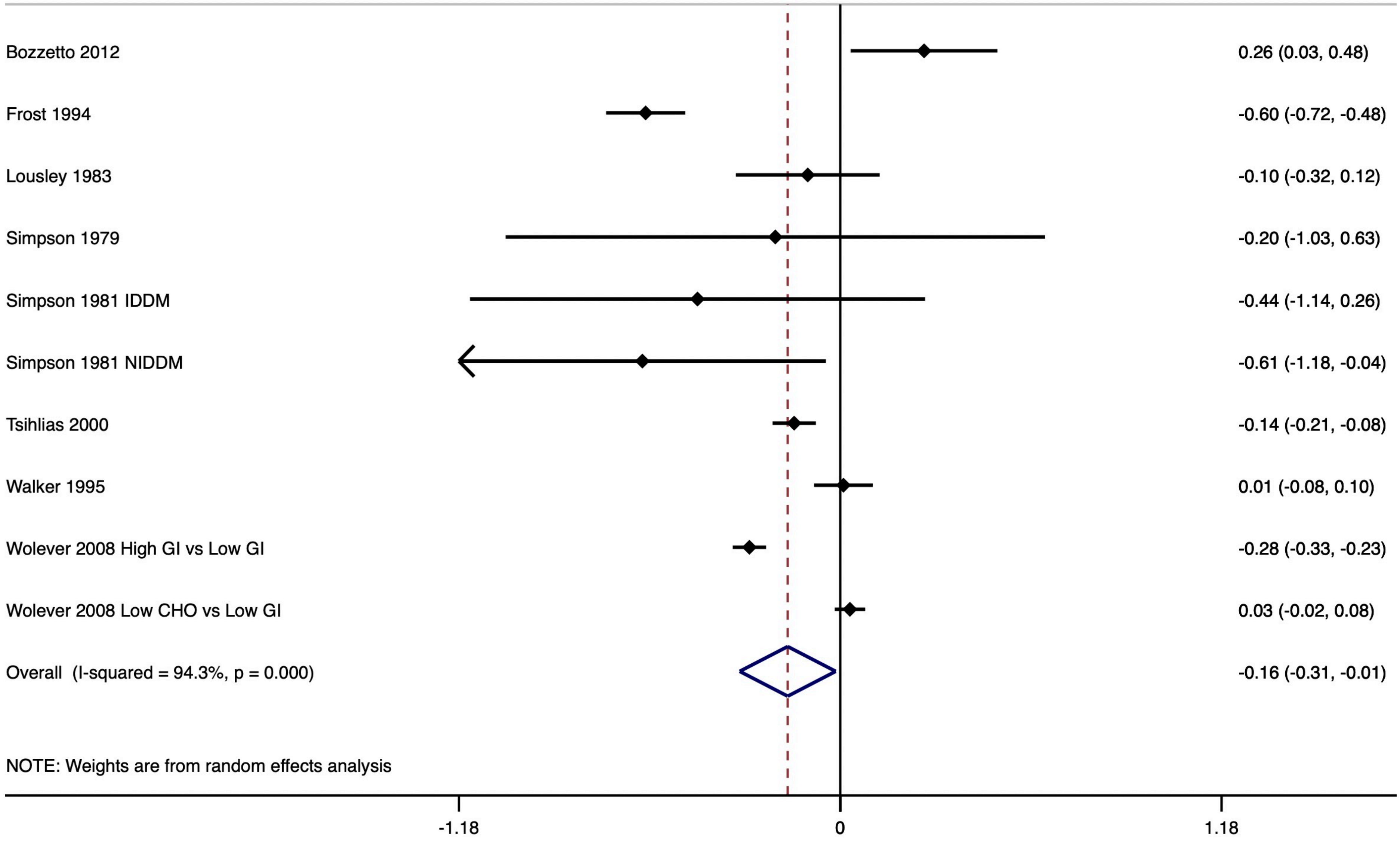


**3.5 LDL cholesterol mmol/L**

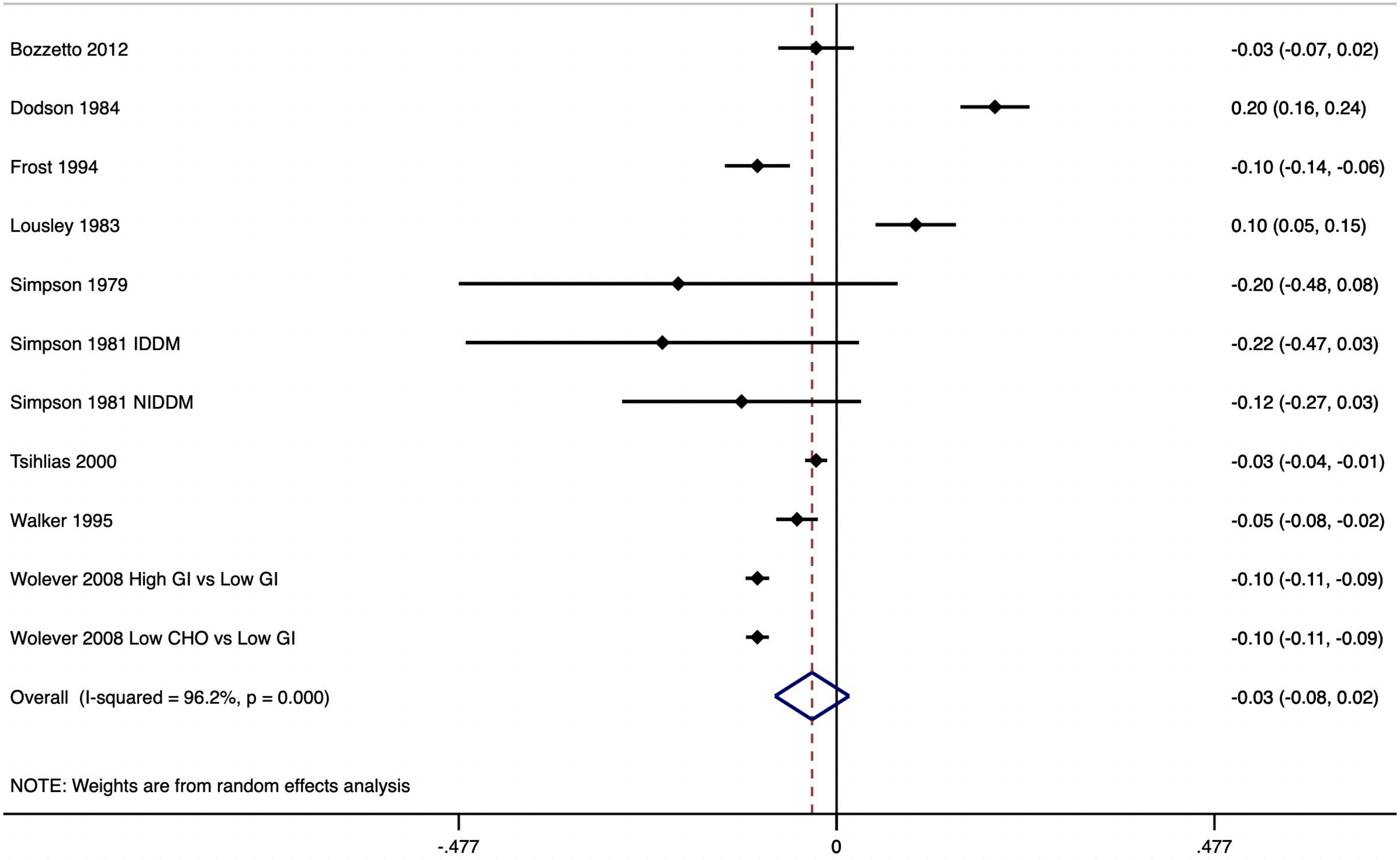


**3.6 HDL cholesterol mmol/L**

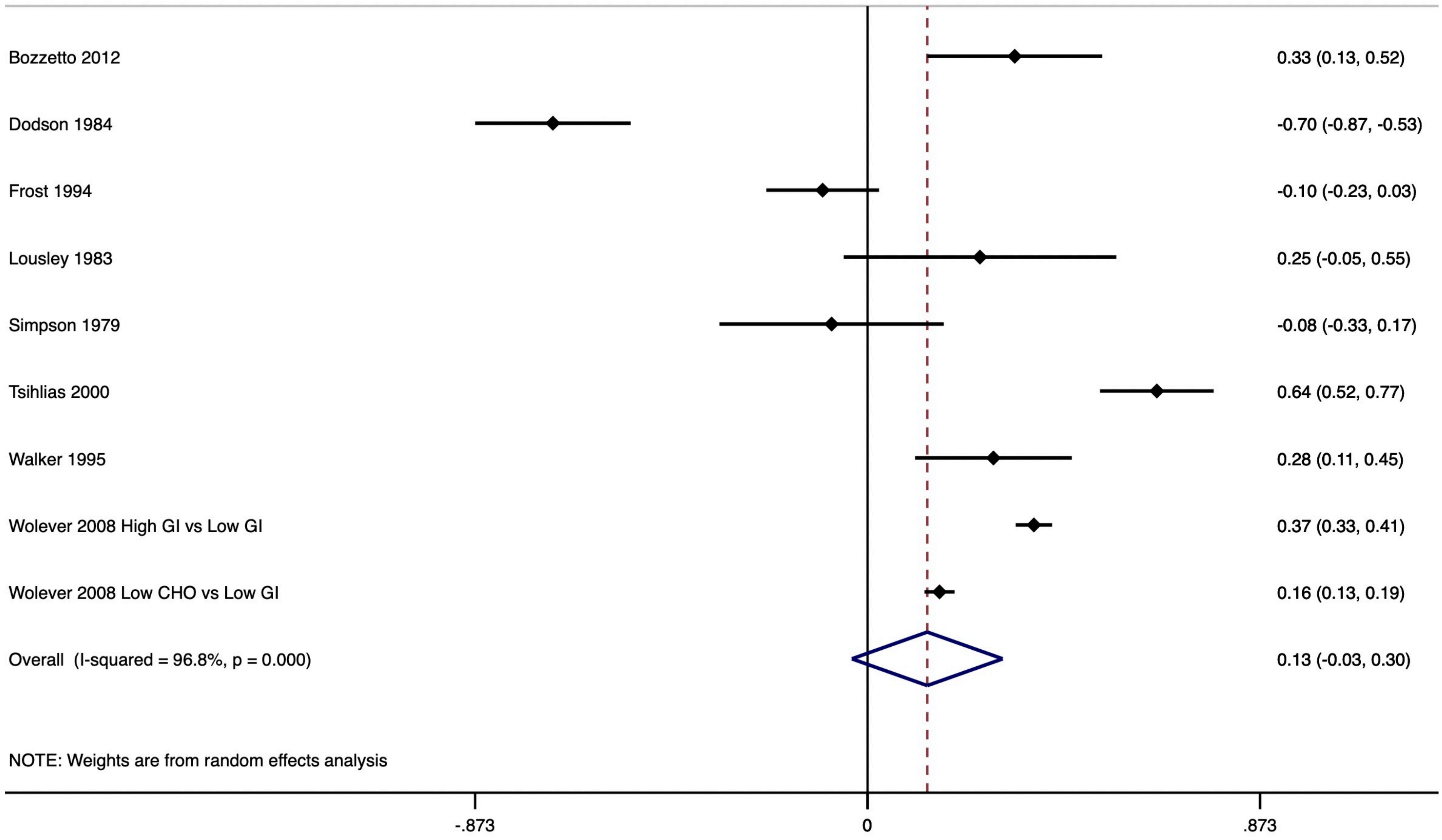


**3.7 Triglycerides mmol/L**

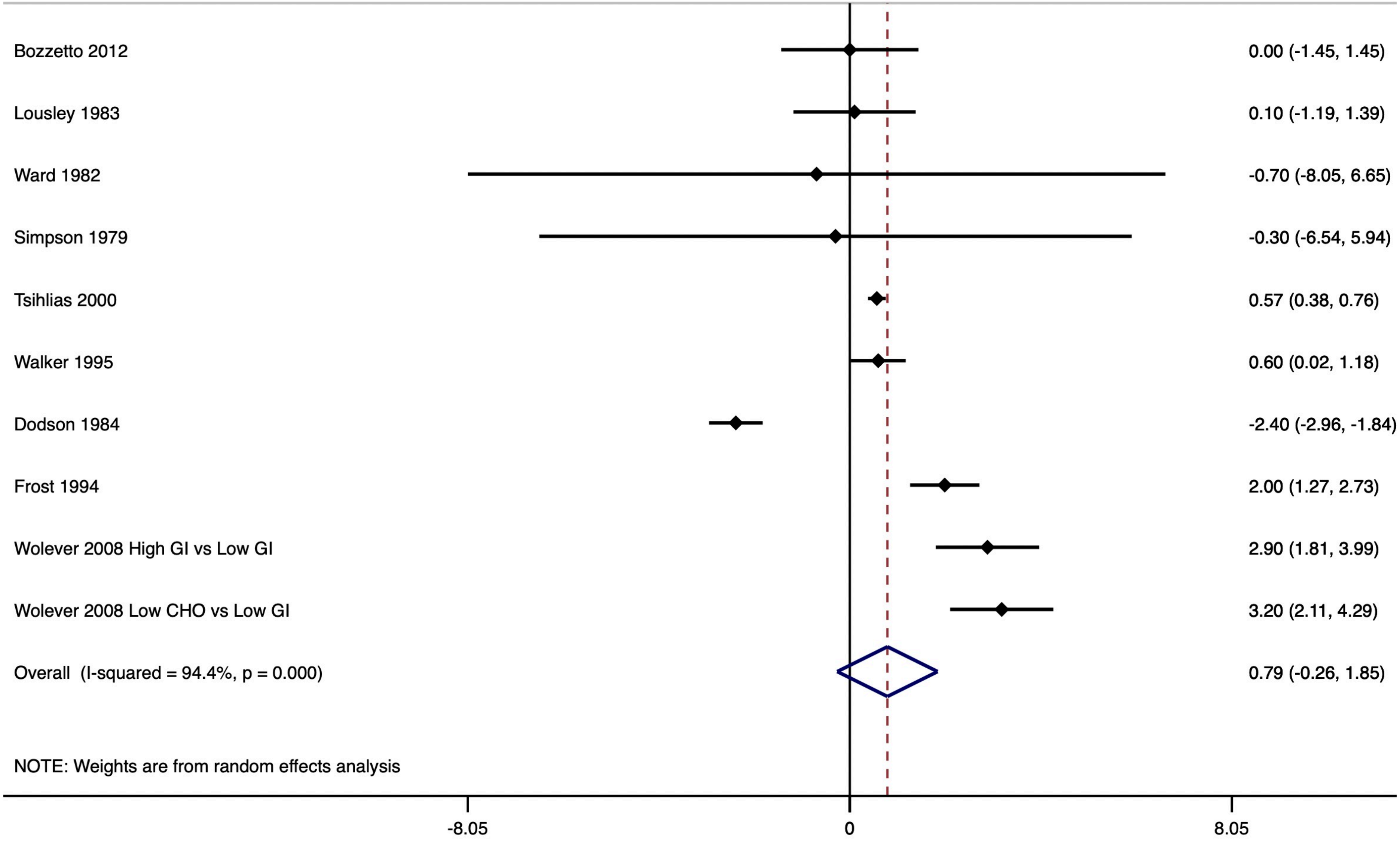


**3.8 Body weight kg**

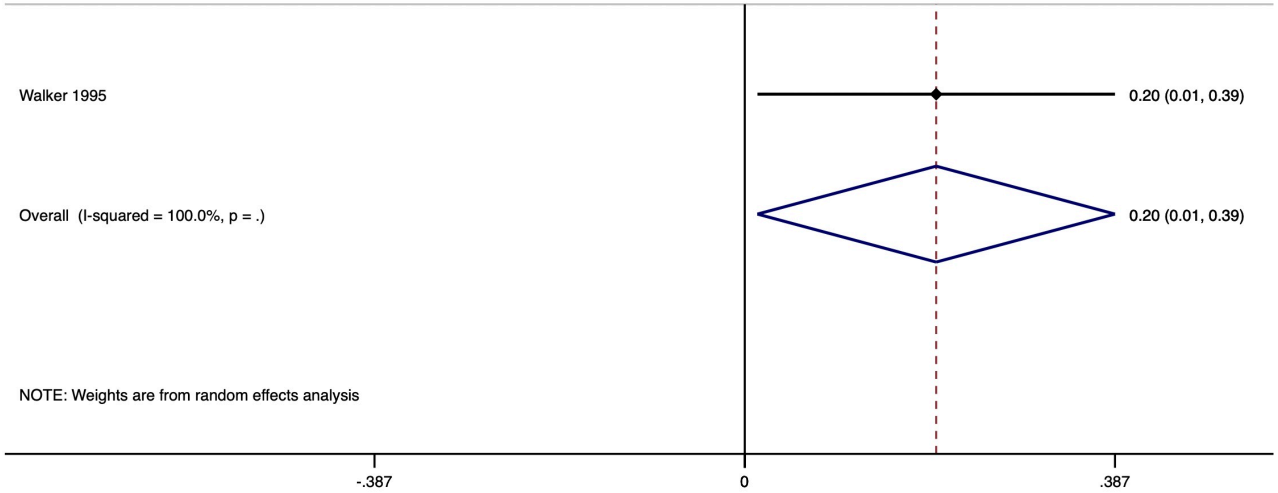
Walker 1995
0.20 (0.01, 0.39)
Overall (I-squared = 100.0%, p = .)
0.20 (0.01, 0.39)
NOTE: Weights are from random effects analysis
-.387
0
.387

### 3.9 BMI

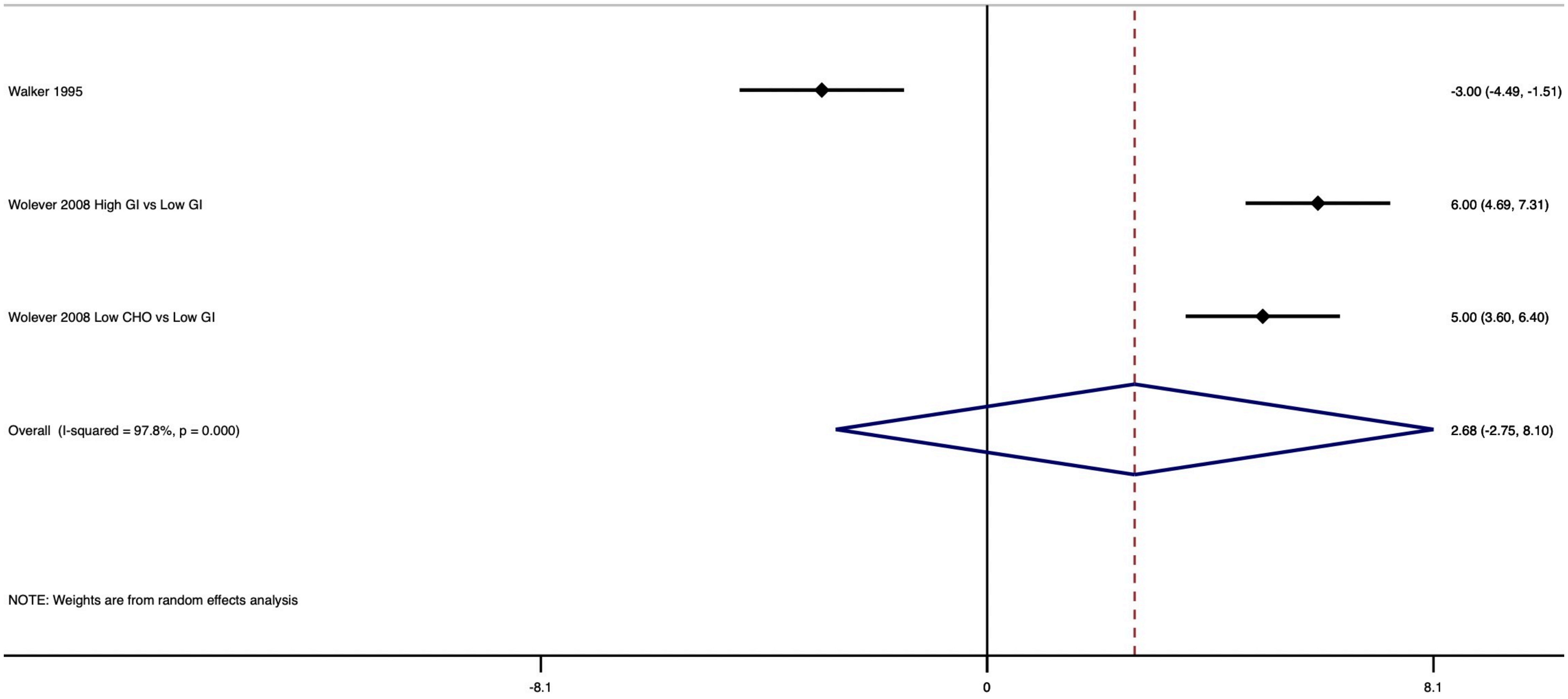
Walker 1995
-3.00 (-4.49, -1.51)
Wolever 2008 High GI vs Low GI
6.00 (4.69, 7.31)
Wolever 2008 Low CHO vs Low GI
5.00 (3.60, 6.40)
Overall (I-squared = 97.8%, p = 0.000)
2.68 (-2.75, 8.10)
NOTE: Weights are from random effects analysis
-8.1
0
8.1

**3.10 SBP mmHg**

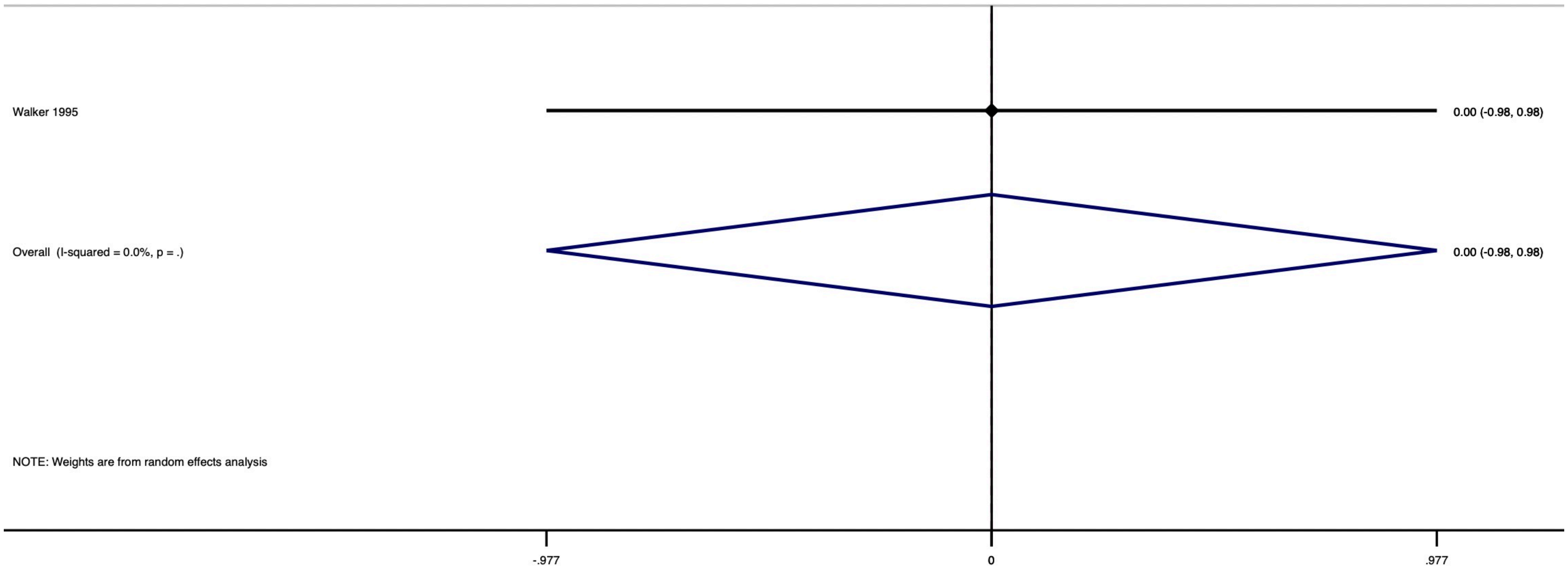
Walker 1995
0.00 (-0.98, 0.98)
Overall (I-squared = 0.0%, p = .)
0.00 (-0.98, 0.98)
NOTE: Weights are from random effects analysis
-.977
0
.977

## 3.11 DBP mmHg

## Supplemental Table 4: Meta-regression analyses per outcome

| Sensitivity analyses | **Body weight** | **HbA1c** | **Fasting glucose** | **Fasting insulin** | **Total cholesterol** | **LDL cholesterol** | **HDL cholesterol** | **Triglycerides** | **SBP** |
|---|---|---|---|---|---|---|---|---|---|
| Egger small study effects | 0.811 | 0.249 | 0.327 | 0.700 | 0.520 | 0.366 | 0.417 | 0.565 | 0.270 |
| Trim and fill analyses | NA | NA | NA | NA | NA | NA | NA | NA | NA |
| Influence analyses | Not detected | Not detected | Not detected | Not detected | Not detected | Not detected | Not detected | Not detected | Not detected |
| Dichotomous MR | | | | | | | | | |
| Parallel or crossover | 0.581 | 0.473 | 0.508 | 0.301 | 0.497 | 0.841 | 0.658 | 0.919 | 0.068 |
| Diabetes type | NA | 0.810 | 0.288 | NA | 0.276 | 0.545 | 0.285 | NA | NA |
| Antihyperglycaemic medication use | **0.009** seven trials where eligible participants could be on anti-hyperglycaemic medication reported a different result (MD -0.25kg (95% CI-1.49 to 0.99)) from that observed in the three trials in participants not on anti-hyperglycaemic drug therapy (MD 2.61kg (95% CI 1.83 to 3.38kg)) | 0.423 | 0.750 | 0.313 | 0.721 | 0.351 | 0.212 | 0.937 | 0.068 |
| All participant insulin use | NA | 0.810 | 0.288 | NA | 0.276 | 0.545 | 0.285 | NA | NA |
| Some participant insulin use | **0.021** Only 1 trial with full insulin use – subgrouping not conducted | 0.185 | 0.288 | NA | 0.526 | 0.545 | 0.121 | NA | NA |

| | | | | | | | | | |
|---|---|---|---|---|---|---|---|---|---|
| Fibre provided as supplement | 0.059 | 0.961 | 0.657 | .999 | 0.319 | 0.230 | 0.101 | 0.857 | NA |
| Isocaloric controlled | 0.440 | 0.981 | 0.848 | 0.313 | 0.773 | 0.401 | 0.720 | 0.686 | 0.068 |
| Intervention was GI advice | 0.051 | 0.062 | 0.508 | 0.301 | 0.494 | 0.841 | 0.310 | 0.195 | 0.068 |
| Intervention was high fibre carbohydrate advice | **0.025** Shown in paper | 0.153 | 0.258 | 0.639 | 0.693 | 0.720 | 0.099 | 0.364 | NA |
| Intervention combined high fibre carbohydrate and unsaturated fat advice | 0.086 | 0.507 | 0.201 | 0.159 | 0.520 | 0.550 | 0.265 | 0.110 | 0.068 |
| Control arm was advice to lower carbohydrates | 0.425 | **0.012** four trials where the control arm promoted low carbohydrate intakes (MD -1.07% -1.86 to -0.28) was greater than the six trials where the control arm did not actively promote lower carbohydrate intakes (MD 0.16% (-0.12 to 0.44)) | 0.133 | 0.655 | 0.241 | 0.890 | 0.519 | 0.126 | 0.732 |
| Control arm was advice to consume unsaturated fats | 0.053 | 0.508 | 0.099 | 0.368 | **0.047** four trials where participants in the lower carbohydrate trial arm received advice to consume unsaturated fats, (MD 0.00 mmol/L (95% | 0.111 | 0.151 | 0.948 | 0.068 |

| | | | | | | | | | |
|---|---|---|---|---|---|---|---|---|---|
| | | | | | CI -0.20 to 0.20). Seven trials where participants in the lower carbohydrate arm did not receive this advice (MD -0.29 mmol/L (95% CI -0.44 to -0.13) | | | | |
| Control arm was advice to consume low fibre carbohydrates | 0.138 | 0.556 | 0.994 | **0.000** Only one trial comparison with control arm advice to consume low fibre carb- subgrouping not conducted | 0.302 | **0.033** eight trials where the lower carbohydrate arm received mixed interventions MD -0.04 (-0.14 to 0.07). two trials where participants in the lower carbohydrate arm received advice to consume simple or low-fibre carbohydrates MD -0.44 (-0.75 to -0.12)) | 0.378 | 0.976 | 0.602 |
| Fibre/Cho ratio <2 | 0.359 | 0.772 | 0.970 | **0.000** three trials with the lowest fibre to carbohydrate increase had a pooled MD 0.02 µIU/mL (95% CI -0.65 to 0.69) | 0.917 | 0.960 | 0.728 | 0.823 | 0.602 |

| | | | | | | | | | |
|---|---|---|---|---|---|---|---|---|---|
| | | | | with an $I^2$ of 18%. The four trials with a higher fibre to carbohydrate increase had a pooled MD -1.40 µIU/mL (95% CI -1.62 to –1.18) and $I^2$ of 0%. | | | | | |
| High CHO had >35g fibre/ day | 0.664 | 0.329 | 0.518 | 0.286 | 0.708 | 0.446 | 0.619 | 0.875 | 0.068 |
| Categorical MR | | | | | | | | | |
| How values per comparison were derived | No | No | No | No | Seven trials with pre post data MD -0.12 (-0.26 to 0.02). 4 trials with webplot or imputed values MD -0.44 (-0.84 to -0.04). Interpretation – large overlap in CI | No | No | No | Too few to run |
| The decade the study published | No | **Studies in 80's different,** however no consistent trend across decades | No | No | No | No | No | No | Too few to run |
| Geographical region studies conducted in | No | **Potential regional differences,** however each driven by the regions represented by only one trial. | **Potential regional differences – six** UK studies MD -1.28 (-1.55 to -1.02) I2 of 0%. Five studies from any other region 0.24 (- | No | No | No | No | No | Too few to run |

| | | | | | | | | | |
|---|---|---|---|---|---|---|---|---|---|
| | | | 0.24 to 0.72) I2 of 97%. | | | | | | |
| Risk of Bias | No | **Bias differences** Five studies with ROB score 1-3 MD -0.86 (-1.87 to 0.14) Five studies with ROB 4-7 MD -0.24 (-0.99 to 0.51). Interpretation – large overlap in CI | No | No | **Bias differences** 6 trials with ROB score 1-3 MD -0.34 (-0.53 to -0.15) 5 trials with ROB score 4-7 MD -0.03 (-0.18 to 0.12) Interpretation – complete overlap in CI | No | **Bias differences** 6 trials with bias 1-3 have MD -0.05 (-0.12 to 0.02) 5 trials with ROB 4-7 have MD -0.02 (-0.09 to 0.05). Interpretation – large overlap in CI | **Bias differences** 4 trials Rob 1-3 MD 0.18 (-0.26 to 0.62) 5 trials ROB 4-7 MD 0.10 (-0.11 to 0.30) Interpretation – complete overlap in CI | Too few to run |
| Continuous MR | | | | | | | | | |
| N participants | No association detected | No association detected | No association detected | No association detected | No association detected | No association detected | No association detected | Too few to run | Too few to run |
| Trial duration in weeks | Too small to run | No association detected | No association detected | Too few to run | Too few to run | No association detected | Too few to run | Too few to run | Too few to run |
| Difference in CHO%TE between intervention and control | No association detected | **Greater the difference, better the result (as reflected in dichotomous MR above)** | **Greater the difference, better the result** | Too few to run | **Greater the difference, better the result** | No association detected | No association detected | Too few to run | Too few to run |
| Difference in fibre g/day between intervention and control | No association detected. Trends to improvements until 25g then flat. | No association detected | **Greater the difference, better the result** | Too few to run | **Greater the difference, better the result** | **Greater the difference, better the result** | No association detected | Too few to run | Too few to run |
| Difference in Fibre:CHO ratio between intervention and control | No association detected. Trends higher fibre to CHO ratio = better result | No association detected. Trends higher fibre to CHO ratio = better result | No association detected. Trends higher fibre to CHO ratio = better result | Too few to run | No association detected. Trends higher fibre to CHO ratio = better result | No association detected. Trends higher fibre to CHO ratio = better result | No association detected | Too few to run | Too few to run |

Note: Dark grey shaded cells with bold text indicate significant results from the dichotomous meta regression

BMI and DBP outcomes had too few trial comparisons to run, so are not shown here. The value shown in cells to three decimal places for the sensitivity analyses and the dichotomous meta regressions are p values. While this single number does not adequately describe

the relationship between regression variable and pooled results, we used a value of <0.050 to determine when subgrouping should or should not be shown. Categorical meta regressions were used to indicate where there might be a trend or pattern worth investigating by subgrouping. Dichotomous or categorical meta regression results where only one trial comparison is identified as different than the remaining pool of studies are not shown given the likelihood that there are other differences in that one trial beyond the single variable under consideration.

## Online Only Supplemental Material References


1. Abbasnezhad, A., et al., *Effect of Different Dietary Approaches in Comparison with High/Low-Carbohydrate Diets on Systolic and Diastolic Blood Pressure in Type 2 Diabetic Patients: A Systematic Review and Meta-Analysis.* Prev Nutr Food Sci, 2020. **25**(3): p. 233-245.
2. Alarim, R.A., et al., *Effects of the Ketogenic Diet on Glycemic Control in Diabetic Patients: Meta-Analysis of Clinical Trials.* Cureus, 2020. **12**(10): p. e10796.
3. Amini, M.R., et al., *Effect of ketogenic diet on blood pressure: A GRADE-Assessed systematic review and meta-analysis of randomized controlled trials.* Nutr Metab Cardiovasc Dis, 2024. **34**(4): p. 823-837.
4. Anderson, J.W., et al., *Carbohydrate and fiber recommendations for individuals with diabetes: a quantitative assessment and meta-analysis of the evidence.* J Am Coll Nutr, 2004. **23**(1): p. 5-17.
5. Apekey, T.A., et al., *Comparison of the Effectiveness of Low Carbohydrate Versus Low Fat Diets, in Type 2 Diabetes: Systematic Review and Meta-Analysis of Randomized Controlled Trials.* Nutrients, 2022. **14**(20).
6. Castañeda-González, L.M., M. Bacardí Gascón, and A. Jiménez Cruz, *Effects of low carbohydrate diets on weight and glycemic control among type 2 diabetes individuals: a systemic review of RCT greater than 12 weeks.* Nutr Hosp, 2011. **26**(6): p. 1270-6.
7. Choi, J.H., et al., *Effect of carbohydrate-restricted diets and intermittent fasting on obesity, type 2 diabetes mellitus, and hypertension management: consensus statement of the Korean Society for the Study of obesity, Korean Diabetes Association, and Korean Society of Hypertension.* Clinical Hypertension, 2022. **28**(1): p. 26.
8. Choy, K.Y.C. and J.C.Y. Louie, *The effects of the ketogenic diet for the management of type 2 diabetes mellitus: A systematic review and meta-analysis of recent studies.* Diabetes Metab Syndr, 2023. **17**(12): p. 102905.
9. Dyson, P., *Very low carbohydrate ketogenic diets and diabetes.* Practical Diabetes, 2020. **37**(4): p. 121-126.
10. Fan, Y., et al. *Effects of low carbohydrate diets in individuals with type 2 diabetes : systematic review and meta-analysis*. 2016.
11. Goldenberg, J.Z., et al., *Efficacy and safety of low and very low carbohydrate diets for type 2 diabetes remission: systematic review and meta-analysis of published and unpublished randomized trial data.* Bmj, 2021. **372**: p. m4743.
12. Hernández Alcantara, G., A. Jiménez Cruz, and M. Bacardí Gascón, *[EFFECT OF LOW CARBOHYDRATE DIETS ON WEIGHT LOSS AND GLYCOSILATED HEMOGLOBIN IN PEOPLE WITH TYPE 2 DIABETES: SYSTEMATIC REVIEW].* Nutr Hosp, 2015. **32**(5): p. 1960-6.
13. Huntriss, R., M. Campbell, and C. Bedwell, *The interpretation and effect of a low-carbohydrate diet in the management of type 2 diabetes: a systematic review and meta-analysis of randomised controlled trials.* Eur J Clin Nutr, 2018. **72**(3): p. 311-325.
14. Jayedi, A., et al., *Dose-dependent effect of carbohydrate restriction for type 2 diabetes management: a systematic review and dose-response meta-analysis of randomized controlled trials.* Am J Clin Nutr, 2022. **116**(1): p. 40-56.
15. Jooste, B.R., et al., *Effectiveness of Technology-Enabled, Low Carbohydrate Dietary Interventions, in the Prevention or Treatment of Type 2 Diabetes Mellitus in Adults: A Systematic Literature Review of Randomised Controlled and Non-Randomised Trials.* Nutrients, 2023. **15**(20).

16. Kirk, J.K., et al., *Restricted-carbohydrate diets in patients with type 2 diabetes: a meta-analysis.* J Am Diet Assoc, 2008. **108**(1): p. 91-100.
17. Kodama, S., et al., *Influence of fat and carbohydrate proportions on the metabolic profile in patients with type 2 diabetes: a meta-analysis.* Diabetes Care, 2009. **32**(5): p. 959-65.
18. Korsmo-Haugen, H.K., et al., *Carbohydrate quantity in the dietary management of type 2 diabetes: A systematic review and meta-analysis.* Diabetes Obes Metab, 2019. **21**(1): p. 15-27.
19. Li, M. and J. Yuan, *Effects of very low-carbohydrate ketogenic diet on lipid metabolism in patients with type II diabetes mellitus: a meta-analysis.* Nutr Hosp, 2022. **39**(4): p. 916-923.
20. Li, S., L. Ding, and X. Xiao, *Comparing the Efficacy and Safety of Low-Carbohydrate Diets with Low-Fat Diets for Type 2 Diabetes Mellitus Patients: A Systematic Review and Meta-Analysis of Randomized Clinical Trials.* Int J Endocrinol, 2021. **2021**: p. 8521756.
21. Luo, W., et al., *Low carbohydrate ketogenic diets reduce cardiovascular risk factor levels in obese or overweight patients with T2DM: A meta-analysis of randomized controlled trials.* Front Nutr, 2022. **9**: p. 1092031.
22. McArdle, P.D., et al., *Carbohydrate restriction for glycaemic control in Type 2 diabetes: a systematic review and meta-analysis.* Diabet Med, 2019. **36**(3): p. 335-348.
23. Meng, Y., et al., *Efficacy of low carbohydrate diet for type 2 diabetes mellitus management: A systematic review and meta-analysis of randomized controlled trials.* Diabetes Res Clin Pract, 2017. **131**: p. 124-131.
24. Nicholas, A.P., et al., *Restricting carbohydrates and calories in the treatment of type 2 diabetes: a systematic review of the effectiveness of 'low-carbohydrate' interventions with differing energy levels.* J Nutr Sci, 2021. **10**: p. e76.
25. Parry-Strong, A., et al., *Very low carbohydrate (ketogenic) diets in type 2 diabetes: A systematic review and meta-analysis of randomized controlled trials.* Diabetes Obes Metab, 2022. **24**(12): p. 2431-2442.
26. Rafiullah, M., M. Musambil, and S.K. David, *Effect of a very low-carbohydrate ketogenic diet vs recommended diets in patients with type 2 diabetes: a meta-analysis.* Nutrition Reviews, 2022. **80**(3): p. 488-502.
27. Sainsbury, E., et al., *Effect of dietary carbohydrate restriction on glycemic control in adults with diabetes: A systematic review and meta-analysis.* Diabetes Res Clin Pract, 2018. **139**: p. 239-252.
28. Silverii, G.A., et al., *Low-carbohydrate diets and type 2 diabetes treatment: a meta-analysis of randomized controlled trials.* Acta Diabetologica, 2020. **57**(11): p. 1375-1382.
29. Skow, S.L. and R.K. Jha, *A Ketogenic Diet is Effective in Improving Insulin Sensitivity in Individuals with Type 2 Diabetes.* Curr Diabetes Rev, 2023. **19**(6): p. e250422203985.
30. Snorgaard, O., et al., *Systematic review and meta-analysis of dietary carbohydrate restriction in patients with type 2 diabetes.* BMJ Open Diabetes Res Care, 2017. **5**(1): p. e000354.
31. Stamati, A., et al., *Efficacy and safety of carbohydrate restriction in patients with type 1 diabetes: A systematic review and meta-analysis.* Diabetes Obes Metab, 2023. **25**(9): p. 2770-2773.

32. Tinguely, D., J. Gross, and C. Kosinski, *Efficacy of Ketogenic Diets on Type 2 Diabetes: a Systematic Review.* Curr Diab Rep, 2021. **21**(9): p. 32.
33. Turton, J.L., R. Raab, and K.B. Rooney, *Low-carbohydrate diets for type 1 diabetes mellitus: A systematic review.* PLoS One, 2018. **13**(3): p. e0194987.
34. Turton, J., et al., *An evidence-based approach to developing low-carbohydrate diets for type 2 diabetes management: A systematic review of interventions and methods.* Diabetes Obes Metab, 2019. **21**(11): p. 2513-2525.
35. Valenzuela Mencía, J., et al., *[Diets low in carbohydrates for type 2 diabetics. Systematic review].* Nutr Hosp, 2017. **34**(1): p. 224-234.
36. van Zuuren, E.J., et al., *Effects of low-carbohydrate- compared with low-fat-diet interventions on metabolic control in people with type 2 diabetes: a systematic review including GRADE assessments.* Am J Clin Nutr, 2018. **108**(2): p. 300-331.
37. Yuan, X., et al., *Effect of the ketogenic diet on glycemic control, insulin resistance, and lipid metabolism in patients with T2DM: a systematic review and meta-analysis.* Nutr Diabetes, 2020. **10**(1): p. 38.
38. Zaki, H.A., et al., *Clinical Assessment of Intermittent Fasting With Ketogenic Diet in Glycemic Control and Weight Reduction in Patients With Type II Diabetes Mellitus: A Systematic Review and Meta-Analysis.* Cureus, 2022. **14**(10): p. e30879.
39. Zaki, H.A., et al., *A Comparative Study Evaluating the Effectiveness Between Ketogenic and Low-Carbohydrate Diets on Glycemic and Weight Control in Patients With Type 2 Diabetes Mellitus: A Systematic Review and Meta-Analysis.* Cureus, 2022. **14**(5): p. e25528.
40. Zhou, C., et al., *Ketogenic Diet Benefits to Weight Loss, Glycemic Control, and Lipid Profiles in Overweight Patients with Type 2 Diabetes Mellitus: A Meta-Analysis of Randomized Controlled Trails.* Int J Environ Res Public Health, 2022. **19**(16).
41. Abboud, M., et al., *Effect of Ketogenic Diet on Quality of Life in Adults with Chronic Disease: A Systematic Review of Randomized Controlled Trials.* Nutrients, 2021. **13**(12).
42. Abbasnezhad, A., et al., *Effect of different dietary approaches compared with a regular diet on systolic and diastolic blood pressure in patients with type 2 diabetes: A systematic review and meta-analysis.* Diabetes Res Clin Pract, 2020. **163**: p. 108108.
43. Aghnezhad, M., R. Vettor, and N. Riedel, *The Ketogenic Diet for Type 2 Diabetes Management.* Obesity Facts, 2024. **17 (Suppl. 1): 7–515**.
44. Ajala, O., P. English, and J. Pinkney, *Systematic review and meta-analysis of different dietary approaches to the management of type 2 diabetes.* Am J Clin Nutr, 2013. **97**(3): p. 505-16.
45. Bajorek, S.A. and C.M. Morello, *Effects of dietary fiber and low glycemic index diet on glucose control in subjects with type 2 diabetes mellitus.* Ann Pharmacother, 2010. **44**(11): p. 1786-92.
46. Bierbaum, M., et al., *Efficacy of diets in the treatment of type 2 diabetes: A systematic review.* Der Diabetologe, 2015. **11**: p. 50-57.
47. Bonekamp, N.E., et al., *Effect of dietary patterns on cardiovascular risk factors in people with type 2 diabetes. A systematic review and network meta-analysis.* Diabetes Res Clin Pract, 2023. **195**: p. 110207.
48. Buccino, J., et al., *Systematic review of the dietary management of children with type 1 diabetes.* Canadian Journal of Diabetes, 2004. **28**: p. 219-225.

49. Castaneda-Gonzalez, L.M., M. Bacardi-Gascon, and A. Jimenez-Cruz, *Long-term effects of low carbohydrate diets on weight, lipids and A1C among type 2 diabetes individuals.* Obesity Reviews, 2011. **12**(s1): p. 63-279.
50. Choi, Y.J., S.M. Jeon, and S. Shin, *Impact of a Ketogenic Diet on Metabolic Parameters in Patients with Obesity or Overweight and with or without Type 2 Diabetes: A Meta-Analysis of Randomized Controlled Trials.* Nutrients, 2020. **12**(7).
51. Churuangsuk, C., et al., *Diets for weight management in adults with type 2 diabetes: an umbrella review of published meta-analyses and systematic review of trials of diets for diabetes remission.* Diabetologia, 2022. **65**(1): p. 14-36.
52. Dening, J., et al., *Web-based interventions for dietary behavior in adults with type 2 diabetes: systematic review of randomized controlled trials.* Journal of medical Internet research, 2020. **22**(8): p. e16437.
53. Emadian, A., et al., *The effect of macronutrients on glycaemic control: a systematic review of dietary randomised controlled trials in overweight and obese adults with type 2 diabetes in which there was no difference in weight loss between treatment groups.* Br J Nutr, 2015. **114**(10): p. 1656-66.
54. Franz, M.J., et al., *The evidence for medical nutrition therapy for type 1 and type 2 diabetes in adults.* J Am Diet Assoc, 2010. **110**(12): p. 1852-89.
55. Harrington, A. and D. Malone, *PDB7 DIET THERAPIES IN PATIENTS WITH TYPE-2 DIABETES: A MIXED-TREATMENT COMPARISON OF RANDOMIZED CONTROL TRIALS.* Value in Health, 2011. **14**(3): p. A92.
56. Jing, T., et al., *Effect of Dietary Approaches on Glycemic Control in Patients with Type 2 Diabetes: A Systematic Review with Network Meta-Analysis of Randomized Trials.* Nutrients, 2023. **15**(14).
57. Jirapinyo, P., et al., *Tu1931-A Comparison of Diet Plan Outcomes in Diabetes Management: A Systematic Review and Meta-Analysis.* Gastroenterology, 2018. **154**(6): p. S-1057-S-1058.
58. Johnson, S.T., et al., *In search of quality evidence for lifestyle management and glycemic control in children and adolescents with type 2 diabetes: A systematic review.* BMC Pediatr, 2010. **10**: p. 97.
59. Lu, K., et al., *Effect of viscous soluble dietary fiber on glucose and lipid metabolism in patients with type 2 diabetes mellitus: a systematic review and meta-analysis on randomized clinical trials.* Front Nutr, 2023. **10**: p. 1253312.
60. Maula, A., et al., *Educational weight loss interventions in obese and overweight adults with type 2 diabetes: a systematic review and meta-analysis of randomized controlled trials.* Diabet Med, 2020. **37**(4): p. 623-635.
61. Mcardle, P.D., P. Gill, and S. Greenfield. *Quantity of Carbohydrate in Type 2 Diabetes: A Systematic Review*. in *DIABETES*. 2017. AMER DIABETES ASSOC 1701 N BEAUREGARD ST, ALEXANDRIA, VA 22311-1717 USA.
62. Naude, C.E., et al., *Low carbohydrate versus isoenergetic balanced diets for reducing weight and cardiovascular risk: a systematic review and meta-analysis.* PloS one, 2014. **9**(7): p. e100652.
63. Naude, C.E., et al., *Low-carbohydrate versus balanced-carbohydrate diets for reducing weight and cardiovascular risk.* Cochrane Database of Systematic Reviews, 2022(1).

64. Neuenschwander, M., et al., *Impact of different dietary approaches on blood lipid control in patients with type 2 diabetes mellitus: a systematic review and network meta-analysis.* European journal of epidemiology, 2019. **34**: p. 837-852.
65. Nield, L., et al., *Dietary advice for treatment of type 2 diabetes mellitus in adults.* Cochrane database of systematic reviews, 2007(3).
66. Nitzke, D., et al., *Increasing dietary fiber intake for type 2 diabetes mellitus management: A systematic review.* World J Diabetes, 2024. **15**(5): p. 1001-1010.
67. Ojo, O., et al., *The Effect of Dietary Glycaemic Index on Glycaemia in Patients with Type 2 Diabetes: A Systematic Review and Meta-Analysis of Randomized Controlled Trials.* Nutrients, 2018. **10**(3).
68. Pan, B., et al., *The impact of major dietary patterns on glycemic control, cardiovascular risk factors, and weight loss in patients with type 2 diabetes: A network meta-analysis.* J Evid Based Med, 2019. **12**(1): p. 29-39.
69. Papamichou, D., D.B. Panagiotakos, and C. Itsiopoulos, *Dietary patterns and management of type 2 diabetes: A systematic review of randomised clinical trials.* Nutr Metab Cardiovasc Dis, 2019. **29**(6): p. 531-543.
70. Pavlidou, E., et al., *Clinical Evidence of Low-Carbohydrate Diets against Obesity and Diabetes Mellitus.* Metabolites, 2023. **13**(2): p. 240.
71. Reynolds, A., et al., *Carbohydrate quality and human health: a series of systematic reviews and meta-analyses.* Lancet, 2019. **393**(10170): p. 434-445.
72. Reynolds, A.N., A.P. Akerman, and J. Mann, *Dietary fibre and whole grains in diabetes management: Systematic review and meta-analyses.* PLoS Med, 2020. **17**(3): p. e1003053.
73. Ross, L.J., et al., *Exploring the highs and lows of very low carbohydrate high fat diets on weight loss and diabetes-and cardiovascular disease-related risk markers: A systematic review.* Nutrition & Dietetics, 2021. **78**(1): p. 41-56.
74. Schwingshackl, L., et al., *Impact of different dietary approaches on glycemic control and cardiovascular risk factors in patients with type 2 diabetes: a protocol for a systematic review and network meta-analysis.* Syst Rev, 2017. **6**(1): p. 57.
75. Schwingshackl, L., et al., *A network meta-analysis on the comparative efficacy of different dietary approaches on glycaemic control in patients with type 2 diabetes mellitus.* Eur J Epidemiol, 2018. **33**(2): p. 157-170.
76. Szczerba, E., et al., *Diet in the management of type 2 diabetes: umbrella review of systematic reviews with meta-analyses of randomised controlled trials.* BMJ Med, 2023. **2**(1): p. e000664.
77. Snelson, M., et al., *Metabolic Effects of Resistant Starch Type 2: A Systematic Literature Review and Meta-Analysis of Randomized Controlled Trials.* Nutrients, 2019. **11**(8).
78. Swedish Council on Health Technology, A., *SBU Systematic Review Summaries*, in *Dietary Treatment of Diabetes: A Systematic Review*. 2010, Swedish Council on Health Technology Assessment (SBU)
Copyright © 2010 by the Swedish Council on Health Technology Assessment.: Stockholm.
79. Swedish Council on Health Technology, A., *Swedish Agency for Health Technology Assessment and Assessment of Social Services (SBU): SBU Systematic Review Summaries*, in *Diets for diabetes: A systematic review and assessment of effects, health economic and ethical aspects*. 2022, Swedish Agency for Health Technology Assessment and Assessment of Social Services (SBU)

Copyright © 2022 by SBU – Swedish Agency for Health Technology Assessment and Assessment of Social Services.: Stockholm.
80. Wheeler, M.L., et al., *Macronutrients, food groups, and eating patterns in the management of diabetes: a systematic review of the literature, 2010.* Diabetes Care, 2012. **35**(2): p. 434-45.
81. Whiteley, C., et al., *Determining Dietary Patterns to Recommend for Type 2 Diabetes: An Umbrella Review.* Nutrients, 2023. **15**(4).
82. Wolfram, T. and F. Ismail-Beigi, *Efficacy of high-fiber diets in the management of type 2 diabetes mellitus.* Endocr Pract, 2011. **17**(1): p. 132-42.
83. Xie, Y., et al., *Effects of soluble fiber supplementation on glycemic control in adults with type 2 diabetes mellitus: A systematic review and meta-analysis of randomized controlled trials.* Clin Nutr, 2021. **40**(4): p. 1800-1810.
84. Xu, B., et al., *Higher intake of microbiota-accessible carbohydrates and improved cardiometabolic risk factors: a meta-analysis and umbrella review of dietary management in patients with type 2 diabetes.* The American Journal of Clinical Nutrition, 2021. **113**(6): p. 1515-1530.
85. Xu, D., et al., *Role of Whole Grain Consumption in Glycaemic Control of Diabetic Patients: A Systematic Review and Meta-Analysis of Randomized Controlled Trials.* Nutrients, 2021. **14**(1).
86. Yamada, S., Y. Kabeya, and H. Noto, *Dietary Approaches for Japanese Patients with Diabetes: A Systematic Review.* Nutrients, 2018. **10**(8).
87. Zakarneh, S., Y. Khial, and R. Tayyem, *Dietary Factors Associated with Glycemic Control in Children and Adolescents with Type 1 Diabetes.* Curr Pediatr Rev, 2023.
88. Zeng, B.-t., et al., *Comparative efficacy of different eating patterns in the management of type 2 diabetes and prediabetes: An arm-based Bayesian network meta-analysis.* Journal of Diabetes Investigation, 2023. **14**(2): p. 263-288.
89. Zhang, Y., et al., *The effectiveness of lifestyle interventions for diabetes remission on patients with type 2 diabetes mellitus: A systematic review and meta-analysis.* Worldviews Evid Based Nurs, 2023. **20**(1): p. 64-78.

**Supplemental Table 5: GRADE Tables**

| Certainty assessment | | | | | | | № of patients | | Effect | | Certainty |
|---|---|---|---|---|---|---|---|---|---|---|---|
| № of studies | Study design | Risk of bias | Inconsistency | Indirectness | Imprecision | Other considerations | Higher CHO higher fibre diets | Lower CHO lower fibre diets | Relative (95% CI) | Absolute (95% CI) | |
| **Body weight (assessed with: kg)** | | | | | | | | | | | |
| 10 | randomised trials | not serious | serious[a] | not serious | serious[b] | none | 198 | 245 | - | MD **0.79 higher** (0.26 lower to 1.85 higher) | ⨁⨁◯◯ Low |
| **BMI** | | | | | | | | | | | |
| 1 | randomised trials | very serious[c] | serious[d] | not serious | not serious | none | 24 | 24 | - | MD **0.2 higher** (0.01 higher to 0.39 higher) | ⨁◯◯◯ Very low |
| **HbA1c (assessed with: %)** | | | | | | | | | | | |
| 10 | randomised trials | not serious | serious[e] | not serious | not serious | none | 191 | 237 | - | MD **0.5 lower** (0.99 lower to 0.02 lower) | ⨁⨁⨁◯ Moderate |
| **Fasting glucose (assessed with: mmol/L)** | | | | | | | | | | | |
| 11 | randomised trials | not serious | serious[f] | not serious | not serious | none | 198 | 252 | - | MD **0.38 lower** (0.83 lower to 0.08 higher) | ⨁⨁⨁◯ Moderate |
| **Fasting insulin (assessed with: µIU/ml)** | | | | | | | | | | | |
| 7 | randomised trials | not serious | not serious[g] | not serious | not serious | none | 119 | 165 | - | MD **0.99 lower** (1.83 lower to 0.915 lower) | ⨁⨁⨁⨁ High |
| **Total cholesterol (assessed with: mmol/L)** | | | | | | | | | | | |

| Certainty assessment | | | | | | | № of patients | | Effect | | Certainty |
|---|---|---|---|---|---|---|---|---|---|---|---|
| № of studies | Study design | Risk of bias | Inconsistency | Indirectness | Imprecision | Other considerations | Higher CHO higher fibre diets | Lower CHO lower fibre diets | Relative (95% CI) | Absolute (95% CI) | |
| 11 | randomised trials | not serious | serious[h] | not serious | not serious | none | 217 | 264 | - | MD **0.16 lower** (0.27 lower to 0.05 lower) | ⊕⊕⊕◯ Moderate |
| **LDL cholesterol (assessed with: mmol/L)** | | | | | | | | | | | |
| 10 | randomised trials | not serious | serious[i] | not serious | not serious | none | 192 | 239 | - | MD **0.16 lower** (0.31 lower to 0.01 lower) | ⊕⊕⊕◯ Moderate |
| **HDL cholesterol (assessed with: mmol/L)** | | | | | | | | | | | |
| 11 | randomised trials | not serious | serious[j] | not serious | not serious | none | 217 | 264 | - | MD **0.03 lower** (0.08 lower to 0.02 higher) | ⊕⊕⊕◯ Moderate |
| **Triglycerides (assessed with: mmol/L)** | | | | | | | | | | | |
| 9 | randomised trials | not serious | serious[j] | not serious | serious[b] | none | 190 | 237 | - | MD **0.13 higher** (0.03 lower to 0.3 higher) | ⊕⊕◯◯ Low |
| **SBP (assessed with: mm Hg)** | | | | | | | | | | | |
| 3 | randomised trials | serious[k] | serious[l] | not serious | serious[m] | none | 78 | 125 | - | MD **2.7 higher** (2.8 lower to 8.1 higher) | ⊕◯◯◯ Very low |
| **DBP (assessed with: mm Hg)** | | | | | | | | | | | |
| 1 | randomised trials | very serious[c] | serious[d] | not serious | not serious | none | 24 | 24 | - | MD **0** (1 lower to 1 higher) | ⊕◯◯◯ Very low |

**CI:** confidence interval; **MD:** mean difference

*Explanations*

a. Both the type of intervention delivered (4 trials promoting high intakes of healthy carbohydrates (MD -0.85kg (CI -2.64 to 0.95 vs. 6 trials promoting low GI or high carbohydrate with good fats (MD 1.70kg (CI 0.77 to 2.64)) and the level of antihyperglycaemic agents (3 trials of participants not on these meds (MD2.61 (CI1.83 to 3.38)) vs. 7 trials that included antihypertensive use (MD -0.25 (CI-1.49 to 0.98)) appeared to influence the pooled result.

b. Confidence intervals contain both a small reduction and appreciable increase in outcome.

c. Only one study reporting data on a small number of participants.

d. Inconsistency unable to be considered with one study.

e. Continuous meta regression analyses identified the greater the difference in CHO%TE the greater the HbA1c improvement for those on higher carbohydrate higher fibre diets. The dichotomous meta regression on the type of control group used in the trial identified differences between whether the control arm did not promote low carbohydrate intakes (4 trials MD 0.16 (CI-0.12 to 0.44) versus those trials where the control arm did promote low carbohydrate intakes (6 trials MD -1.07 (-1.86 to -0.28).

f. Continuous meta regression analyses identified the greater the difference in CHO%TE and the greater the difference in fibre intakes between the two intervention arms, the greater the improvement in fasting glucose.

g. Although the initial heterogeneity was high at 93%, a meta regression on the fibre increase relative to the carbohydrate increase identified a difference between the pooled trials. The three trials with the lowest fibre to carbohydrate increase had a pooled MD 0.12 (-3.89 to 4.13) with an I2 of 18%. The four trials with a higher fibre to carbohydrate increase had a pooled MD -8.40 (CI -9.7 to -7.1) and I2 of 0%. Because of the broad nature of our research question, and the identification of an important variable and the resulting low heterogeneity of the subgroup analyses, this has not been downgraded for inconsistency.

h. Continuous meta regression analyses identified the greater the difference in CHO%TE and the greater the difference in fibre intakes between the two intervention arms, the greater the improvement in total cholesterol. There was also a difference in outcome for trials where the low carbohydrate control group received advice to eat unsaturated fats or not. The 4 trials where the lower CHO arm got advice to eat unsaturated fats did not show a difference between intervention arms (MD 0.0 (CI -0.19 to 0.20). There were however appreciable improvements in total cholesterol with higher carbohydrate higher fibre diets in the 7 trials where the lower carbohydrate arm did not get advice to consume unsaturated fats (MD -0.29 (CI-0.44 to -0.13).

i. Continous meta regression indicated that the fibre difference might drive the observed improvement in LDL cholesterol, with higher fibre interventions having greater benefit. The 8 trials control groups with mixed interventions had a MD -0.04 (CI-0.14 to 0.07) while the two interventions that had control arms of low quality carbohydrate had a much larger benefit in LDL cholesterol MD -0.44 (CL-0.75 to -0.12).
j. No meta regression undertaken could explain the high initial heterogeneity.
k. There were only 3 data points available from two trials. Furthermore, influence analysis indicated the results of one trial differed greatly from the two data points available for this outcome from another trial.
l. There were insufficient diversity in data points to meaningfully consider the high initial heterogeneity.
m. Confidence intervals include both a meaningful reduction and a very high increase in outcome.
